# Industrial and Laboratory Technologies for the Chemical Recycling of Plastic Waste

**DOI:** 10.1021/acscatal.4c03194

**Published:** 2024-08-05

**Authors:** Mason T. Chin, Tianning Diao

**Affiliations:** Department of Chemistry, New York University, 100 Washington Square East, New York, New York 10003, United States

**Keywords:** plastic waste, recycling, SPI codes, polymer degradation, catalytic hydrogenolysis

## Abstract

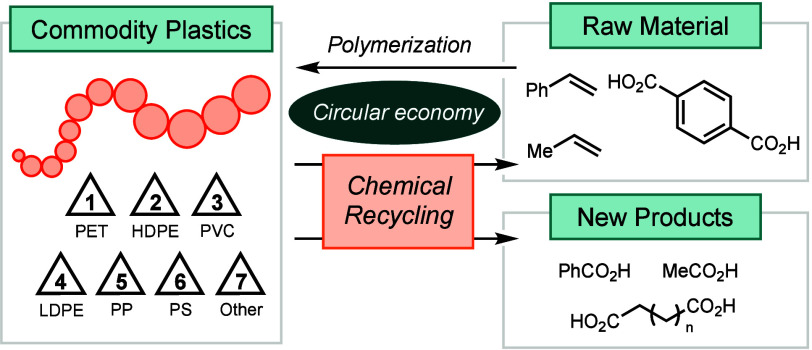

Synthetic polymers play an indispensable role in modern society, finding applications across various sectors ranging from packaging, textiles, and consumer products to construction, electronics, and industrial machinery. Commodity plastics are cheap to produce, widely available, and versatile to meet diverse application needs. As a result, millions of metric tons of plastics are manufactured annually. However, current approaches for the chemical recycling of postconsumer plastic waste, primarily based on pyrolysis, lag in efficiency compared to their production methods. In recent years, significant research has focused on developing milder, economically viable methods for the chemical recycling of commodity plastics, which involves converting plastic waste back into monomers or transforming it into other valuable chemicals. This Perspective examines both industrial and cutting-edge laboratory-scale methods contributing to recent advancements in the field of chemical recycling.

## 1 Accumulation of Plastic Waste from Commodity Polymers and Recycling Strategies

**Figure 1:**
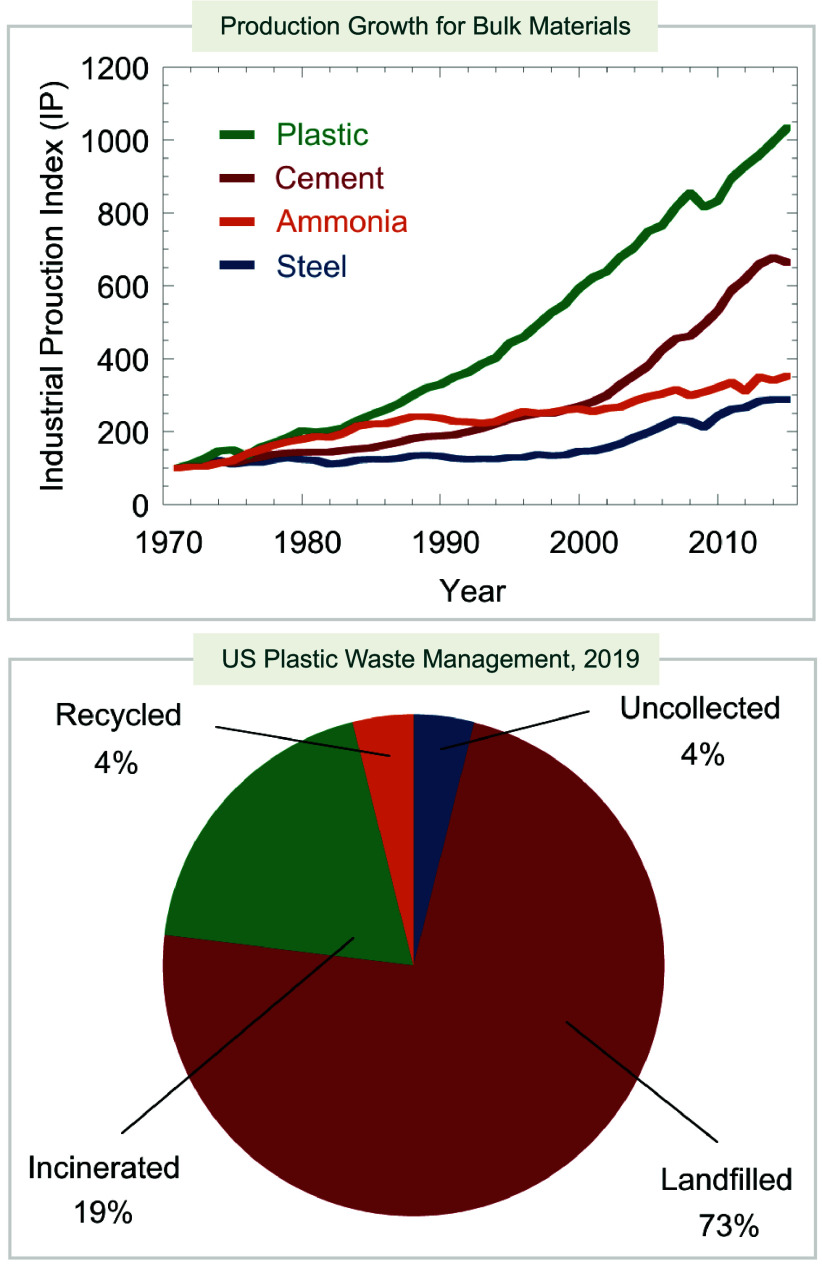
Production of commodity plastics and management of plastic waste.^2^

Since their foundational commercial production in the 1950s, synthetic plastics have experienced enormous growth, attributed to low production costs, versatile applications, and widespread accessibility.^1^ According to a 2018 report by the International Energy Agency (IEA), the demand for commodity plastics has outpaced that of other bulk materials, such as cement and ammonia, with almost a doubling in demand since 2000 (Figure 1).^2^ The production of key commodity plastics will increase from 322 million metric tons (Mt) to 590 Mt by 2050, marking a 45% increase over three decades. As the global consumption of commodity plastics rises, the accumulation of plastic waste is expected to escalate correspondingly. A report by the Organization for Economic Co-operation and Development (OECD) highlights that, as of 2019, only 4% of plastic waste is recycled in the United States, with the global recycling rate estimated to be a mere 9%.^3^

There are four categories of recycling: primary, secondary, tertiary, and quaternary.^4^ Primary recycling, also known as closed-loop recycling, simply reuses postconsumer plastics for the same purpose as the original material without significantly altering its chemical and structural composition. For example, postconsumer plastics from water bottles is transformed into new plastic bottles. Secondary recycling, often referred to as mechanical recycling, processes a single stream of postconsumer plastic into resins, fibers, or sheets, with expanded applications. Due to its cost-effectiveness and simplicity, mechanical recycling is the most commonly practiced; however, this approach often results in materials of lower quality due to the partial degradation occurring during high-temperature reprocessing. The gradual degradation not only limits the potential applications of recycled plastics but also shortens their lifecycle. In addition, mechanical recycling necessitates rigorous sorting of plastic waste streams since only a single type of plastic can be reprocessed at a time.

On the other hand, quaternary recycling entails the combustion of plastic waste to produce energy, a method falling short of sustainability goals. Finally, tertiary recycling, known as chemical recycling, converts plastic waste into valuable chemicals or monomers, which can then be reformed into high-quality materials akin to original materials. Chemical recycling to monomers promotes a circular economy by allowing the continual reuse of materials, which has the added advantage of tolerating other additives and contaminants and potentially reducing the need for extensive sorting associated with mechanical recycling.^5^ Given these advantages, significant research is being directed toward improving the efficiency and cost-effectiveness of chemical recycling to provide a more sustainable strategy for plastic waste management.

## 2 Thermodynamic Considerations of Depolymerization

Polymerization is typically driven by a negative enthalpy change (Δ*H*°), while depolymerization is driven by a positive entropy change (eq 1). For example, in an exergonic olefin polymerization (Δ*G*° < 0), the cleavage of a C–C π-bond and formation of a C–C σ-bond lead to a negative Δ*H*°, whereas Δ*S*° is largely negative due to a decrease in the system’s degree of freedom.^6^ Increasing the temperature enhances the contribution of entropy and shifts Δ*G*°. The temperature at which Δ*G*° = 0, where the rates of polymerization and depolymerization are equal, is known as the ceiling temperature (*T*_c_). The equilibrium favors polymerization when *T* < *T*_c_ and favors depolymerization when *T* > *T*_c_. Several factors can influence *T*_c_, including concentration, pressure, solvent, and monomer structure, all of which should be considered when comparing *T*_c_ values of different polymers.^6−10^ Additionally, the magnitude of the ratio between Δ*H*° and Δ*S*° provides insight into the difference in temperature from *T*_c_ required to theoretically achieve complete polymerization or depolymerization.^5^
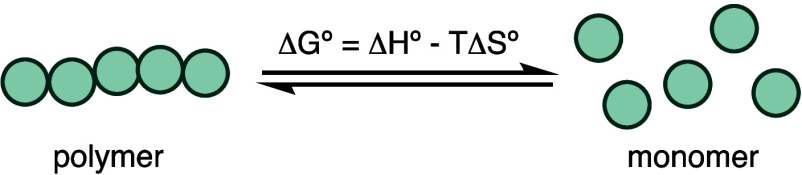
1

Despite the importance of ceiling temperature (*T*_c_) in dictating polymerization/depolymerization equilibria, exploiting *T*_c_ alone to promote depolymerization is not effective for highly exergonic reactions such as ethylene polymerization or vinyl chloride polymerization.^5^ For polyethylene, thermolytic conditions result in byproducts such as long-chain waxes and other gases, achieving only moderate selectivity for ethylene.^11^ Similarly, the thermolysis of poly(vinyl chloride) can undergo side reactions before producing the monomer.^12^ This report discusses state-of-the-art approaches for achieving chemical recycling to monomer for these types of plastics, as well as other commodity plastics.

## 3 Commodity Plastics and Their SPI Codes

**Figure 2:**
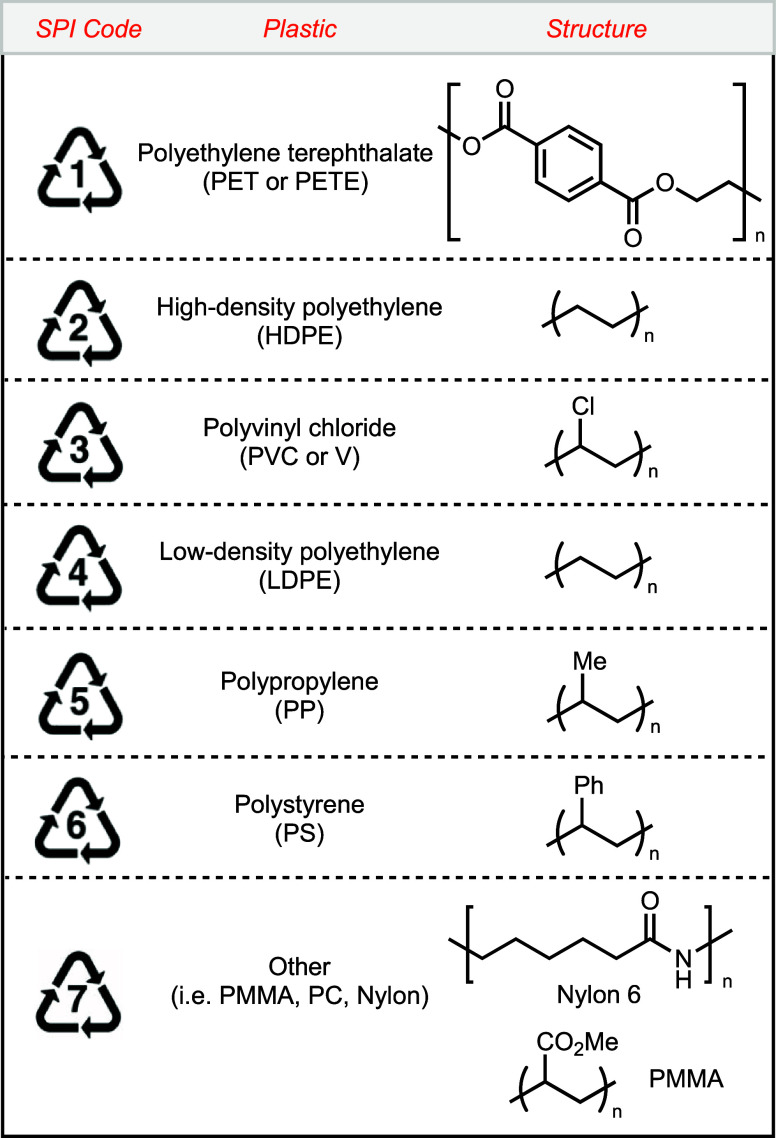
List of plastics and their SPI Codes.

Each type of commodity plastic is assigned a unique identification code by the Society of the Plastic Industry, known as the SPI code. These SPI codes serve to distinguish the various types of common plastics, providing essential information to consumers about their recyclability. There are seven SPI codes designated for the most common commodity plastics, which are tabulated in Figure 2.^13^ This article discusses industrial and state-of-the-art lab methods for the chemical recycling of plastics corresponding to each SPI code number.

### 3.1 Chemical Recycling of Poly(ethylene terephthalate) (PET, SPI Code 1)

Poly(ethylene terephthalate) (PET or PETE) is a polyester thermoplastic widely used in the beverage industry, serving as the primary material for packaging of bottled water, carbonated soft drinks, energy drinks, tea, and coffee. The United Nations University Institute for Water, Environment and Health reported that the estimated annual production of PET bottles in 2019 exceeded 600 billion units, equivalent to approximately 24 million tons of postconsumer PET waste.^14^ PET is marked with an SPI Code of 1, indicating its recyclability and encouraging consumers to participate in curbside recycling programs. In fact, PET is the most recycled plastic among commodity plastics, with approximately 29% of postconsumer PET waste being recycled in 2018.^15^ In commercial settings, PET is predominantly recycled mechanically into fibers which are further processed into products such as carpets, clothing, and other textiles. However, this mechanical recycling process, while economically viable, involves high temperatures for extruding PET into fibers. The forcing conditions lead to undesired degradation of PET through hydrolysis of the ester backbone, which decreases the molecular weight and reduces its quality. This degradation exacerbates with each repeated processing cycle.^16^

**Scheme 1:**
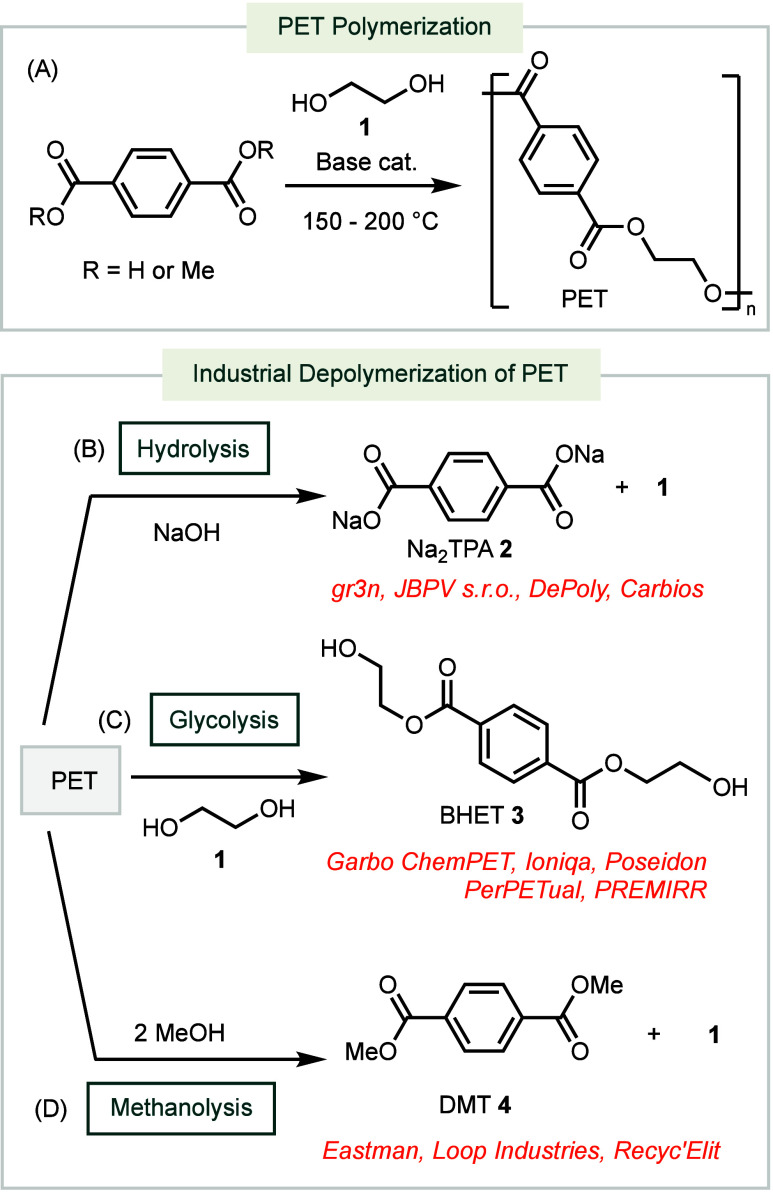
Industrial PET Polymerization and Depolymerization

PET is typically synthesized through the condensation of terephthalic acid or dimethyl terephthalate with ethylene glycol **1**, in the presence of a basic catalyst under heating conditions (Scheme 1A).^17^ Consequently, many industrial chemical recycling methods leverage the reverse process, hydrolysis, to recover the monomers.^18,19^ These recycling methods are categorized into three main types: hydrolysis (Scheme 1B), glycolysis (Scheme 1C), and methanolysis (Scheme 1D).

Industrial hydrolysis of PET typically uses NaOH as a base catalyst due to its low cost, fast reaction rates, and high efficiency compared to other base-mediated depolymerization conditions (Scheme 1B).^20^ In these reactions, base-catalyzed hydrolysis of PET yields the disodium salt of terephthalic acid **2**, which can be conveniently separated from insoluble reaction residues *via* filtration. Microwave irradiation has been employed to accelerate PET depolymerization, with a patent by GR3N reporting a 98% yield of terephthalic acid after just a 15 min residence time.^21−23^ Other patented processes by DePoly use UV irradiation with a TiO_2_ photocatalyst to promote hydrolysis. Additionally, enzymatic methods, such as those developed by Carbios, employ a modified leaf-branch compost cutinase (LCC) enzyme, proven to process up to 2 tons per batch, equivalent to approximately 100,000 bottles per cycle.^24−26^

Glycolysis conditions for converting PET into BHET **3** involve the use of ethylene glycol as the nucleophile to cleave the ester backbone (Scheme 1C). Patents from the industry have documented the use of Lewis acidic catalysts such as Zn(OAc)_2_ to promote the ester exchange of PET with an excess of ethylene glycol.^27−31^ Additionally, Ioniqa Technologies has developed a catalyst comprising an ionic liquid [Bmim]FeCl_4_, tethered to a magnetite nanoparticle via a bridging silane linker.^32^ By 2019, Ioniqa had implemented this innovative technology in a 10-kiloton recycling plant dedicated to the chemically recycling of postconsumer PET.^33^

The industrial methanolysis of PET employs methanol to hydrolyze the ester bonds within the polymer chain to afford **1** and **4**, which can be purified through distillation (Scheme 1D). Patents from Loop Industries report high conversions to **4** at relatively mild temperatures between 60 and 100 °C, in the presence of cosolvents such as DCM and DMSO.^34−37^ Additionally, Recyc’Elit has developed patented conditions for low-temperature methanolysis, utilizing a mixture of alkoxide and amidine base catalysts alongside organic solvents.^38^ Another approach, vapor methanolysis, involves the feeding of methanol vapor into a reactor, allowing for the simultaneous collection of monomers through distillation during the reaction. Eastman Chemical has patented technologies based on vapor methanolysis and has announced commercialization plans to establish a facility with a 100,000 t capacity.^39,40^

Extensive research in academic settings has focused on improving these commercial technologies.^41−43^ More recently, significant efforts have been devoted to advancing enzymatic hydrolysis conditions, relevant to the technology reported by Carbios.^44^ Enzymes provide selectivity for PET within a mixed plastic waste stream, offering a clear advantage. However, challenges persist, such as the high costs associated with enzyme production and the separation of products postdepolymerization, which are more straightforward under traditional PET processing conditions.

### 3.2 Chemical Recycling of High-Density and Low-Density Polyethylene (HPDE, SPI Code 2; LDPE, SPI Code 4)

Polyethylene (PE) is a fully saturated thermoplastic and the most produced plastic worldwide. The two most prevalent types of PE are high-density polyethylene (HDPE), identified by an SPI code of 2, and low-density polyethylene (LDPE), identified by an SPI code of 4. HDPE is produced using Ziegler–Natta catalysts, including heterogeneous titanium complexes with triethylaluminum activators, as well as homogeneous group 4 metallocenes with methylaluminoxane activators.^45,46^ Applying low pressure ethylene results in linear structures with minimal branching.^47^ HDPE is highly crystalline and stiff, ideal for manufacturing bottles, buckets, pipes, and other containers. In contrast, the synthesis of LDPE with radical polymerization under high pressures of ethylene leads to irregular structures with significant branching.^48^ This gives LDPE low crystallinity and high flexibility, suitable for applications in disposable bags, food packaging, and wrapping films.

As of 2019, the annual production of PE in the United States reached approximately 23 million metric tons.^49^ Despite its widespread utilities, only about 930 thousand tons of PE were recycled in the United States.^50^ The mechanical recycling of PE, depending on extrusion conditions, can lead to either oxidation of the carbon backbone or extensive cross-linking and branching. Both alterations occur through hydrogen abstraction mechanisms in the presence of oxygen.^51,52^ These structural modifications can gradually degrade the quality of the recycled PE material over repeated extrusion processes.

**Scheme 2:**
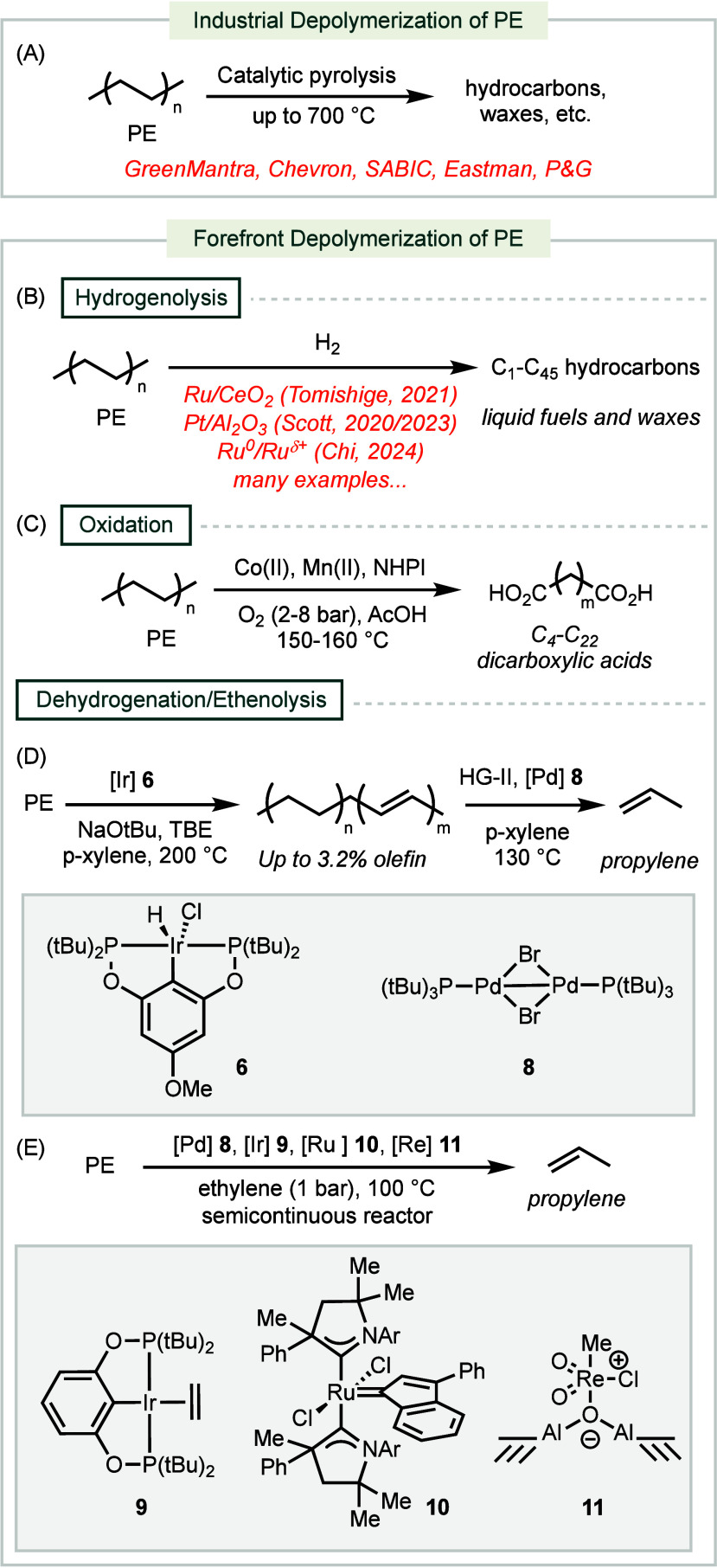
Industrial and Forefront Methods for PE Chemical Recycling

Industry patents for the chemical recycling of PE, held by companies such as Chevron, SABIC, and Proctor & Gamble, employ pyrolytic conditions in catalytic reactors to produce complex mixtures of hydrocarbons (Scheme 2A).^53−57^ For example, GreenMantra Technologies has developed a [Fe–Cu–Mo-P] catalyst supported on aluminum oxide to convert postconsumer PE waste into commercially relevant C_14_–C_41_ waxes and greases.^58^ These pyrolysis conditions, however, are extremely energy intensive, often requiring high temperatures and pressures.

Several state-of-the-art methods for the chemical recycling of PE use hydrogenolysis conditions to fragment the carbon backbone into gas, liquid fuels, and wax products with chain lengths ranging from C_1_ to C_45_ (Scheme 2B).^59^ Heterogeneous transition metal catalysts based on Ru, Zr, Rh, Pt, and Co are typically employed at high temperatures and elevated pressures of hydrogen gas. These reactions may proceed either through σ-bond metathesis or β-alkyl transfer to achieve C–C bond cleavage of PE. For example, a recent study utilizes a heterogeneous Pt/γ-Al_2_O_3_ catalyst to convert PE waste into long-chain alkylbenzene products, which are crucial feedstocks for detergent manufacturing.^60^ Additionally, commercial Ru/C catalysts modified with ethylene glycol promote the formation of Ru^δ+^ species, which performs σ-bond metathesis with PE five times more efficiently than traditional Ru/C catalysts.^61^

Alternative PE depolymerization strategies include oxidation (Scheme 2B). Catalytic systems consisting of Co(II), Mn(II), and NHPI (NHPI = *N*-hydroxyphthalimide) can convert PE into lower molecular weight dicarboxylic acids.^62,63^ NHPI serves as an initiator by abstracting hydrogen atoms on PE, leading to the formation of carbon radicals. The radicals are trapped by O_2_ to form a peroxide intermediate, which is then homolyzed to an alkoxy radical through the Co(II)/Mn(II) catalytic system. β-scission of the alkoxy radical, followed by further oxidation, affords the lower molecular weight carboxylic acid products. These conditions have also been demonstrated to depolymerize PET and polystyrene (PS) into terephthalic acid and benzoic acid, respectively. Additionally, oxidative depolymerization of PE to dicarboxylic acids applies a heterogeneous Ru/TiO_2_ catalyst.^64^

More recently, an alternative approach employs a two-step dehydrogenation/ethenolysis reaction to convert PE waste into propylene (Scheme 2D).^65^ The first dehydrogenation step employs **6**, an Ir hydride complex supported with a PCP pincer ligand, to incorporate up to 3.2% of internal alkene within the backbone of PE. This material is amenable to ethenolysis using the second generation Hoveyda-Grubbs catalyst (HG-II), in conjunction with **8**, an alkene isomerization catalyst, under 25 bar of ethylene gas. The overall process can produce up to 80% yield of propylene from both HDPE and LDPE. Another tandem strategy that combines dehydrogenation, metathesis, and isomerization, uses dehydrogenation catalyst **9**, olefin metathesis catalyst **10**, and heterogeneous isomerization catalyst **11**. This method is implemented in a semicontinuous reactor to convert PE into propylene with up to 94% selectivity.^66^

Although the leading methods are milder and more selective than those currently used in industry, the complexity of these reactions may render them impractical for commercial applications. Moreover, the use of some noble transition metal catalysts may increase the cost of the process. Nevertheless, these advances represent significant achievements in progressing toward a circular economy through postconsumer PE waste management.

### 3.3 Chemical Recycling of Poly(vinyl chloride) (PVC, SPI Code 3)

Poly(vinyl chloride) (PVC) is a high-strength thermoplastic and the third most produced plastic worldwide. Approximately 44 million metric tons of PVC were produced globally in 2018, with projections indicating a 35% increase by 2025.^67^ PVC is extensively used in the construction industry for window and door profiles, as well as drinking and wastewater piping, but it also finds applications in the automobile and packaging industries.^68^ Suspension polymerization is the most widely used commercial method to synthesize PVC.^69^ This process involves agitating a biphasic mixture of water and vinyl chloride, where vinyl chloride is polymerized using a monomer-soluble free radical initiator within aqueous droplets. As the polymerization progresses, PVC precipitates, facilitating isolation.

PVC, identified by an SPI code of 3, should not be mixed with other recyclable plastics due to its incompatibility during the recycling process. This incompatibility largely stems from additives used to enhance thermal and UV stability, including heavy metal stabilizers such as tin, cadmium, and lead.^70^ These additives also complicate the mechanical recycling of postconsumer PVC, necessitating their removal prior to recycling. Advanced sorting methods and separation techniques are available to separate PVC from other plastic waste and additives, but these processes are often costly and labor-intensive.^71,72^ Consequently, despite being the third most produced plastic globally, PVC has the lowest recycling rate among commodity plastics.^41^ Typically, 82% of postconsumer PVC waste is treated through landfilling, and 15% through incineration.^73^

**Scheme 3:**
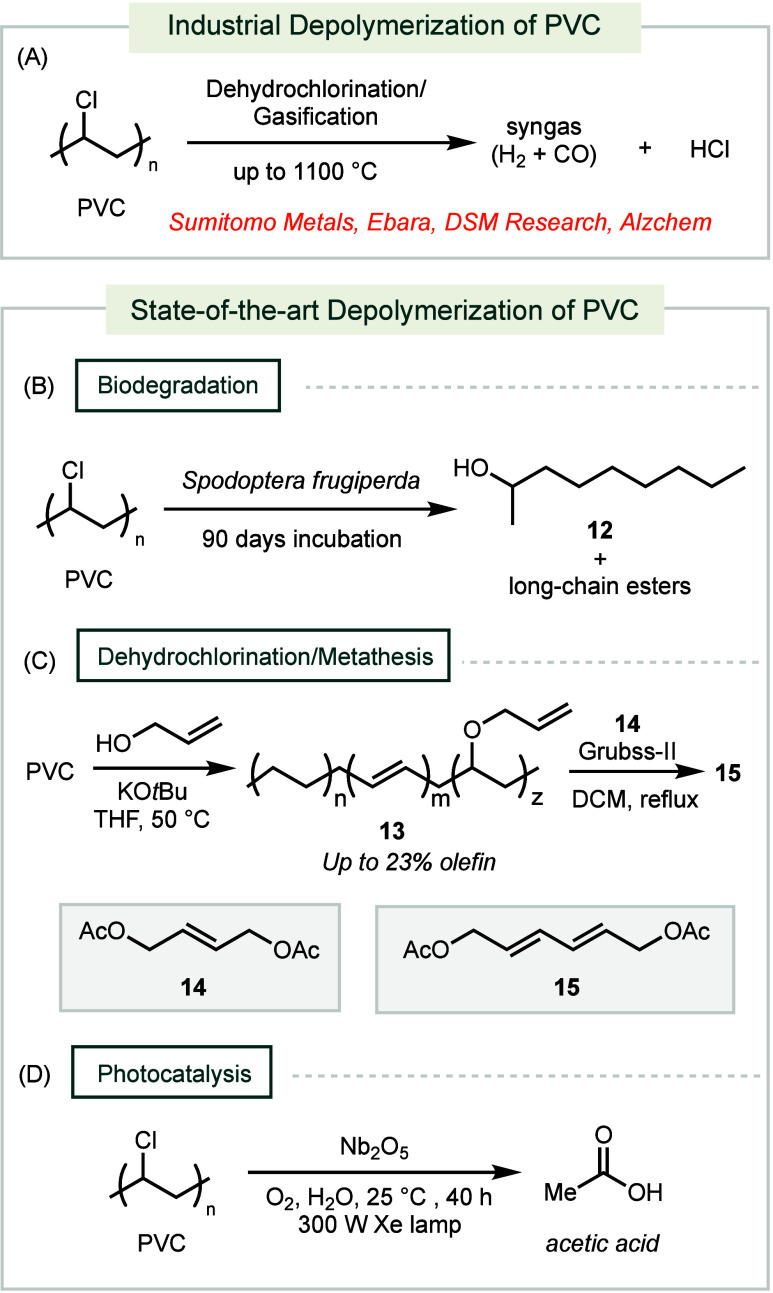
Industrial and Forefront Methods for PVC Depolymerization

Unlike other commodity plastics, PVC is generally not recommended for direct pyrolysis due to the emission of HCl gas and other hazardous chlorine-containing products, which have hindered its commercial-scale processing.^74^ Consequently, alternative chemical recycling strategies have been developed (Scheme 3A). Gasification involves treating PVC with controlled amounts of oxygen or steam under high temperatures to produce CO_2_, syngas (CO and H_2_), and HCl. The primary advantage of gasification is that it converts nearly all of the chlorine content in PVC waste into water-soluble HCl.^67^ Currently, two companies in Japan, Sumitomo Metals and Ebara, operate commercial-scale PVC recycling plants using gasification methods.^75,76^ Additionally, dehydrochlorination processes have been developed to remove chlorine from PVC waste before gasification or pyrolysis.^77^ For example, DSM Research in The Netherlands has developed the REDOP process to dechlorinate mixed plastic waste containing PVC.^78^ The generated HCl is quenched using a water-soluble base, and the dechlorinated plastic waste is recovered as insoluble granules. In Germany, Alzchem operates a plant that converts mixed plastic waste streams containing PVC into syngas, calcium carbide, and HCl, which is then recovered as an aqueous solution.^79^

State-of-the-art methods have been developed to provide milder and more sustainable conditions for chemically recycling PVC. For example, the biodegradation of PVC has been reported through larvae consumption using a strain of *Spodoptera frugiperda* that can consume PVC films as its sole energy source (Scheme 3B).^80,81^ After an incubation period of 90 days, a biofilm formed on the surface of the PVC film, containing a mixture of oxidation products, including **12** and other long-chain esters, as detected by GC-MS analysis. A catalase-peroxidase enzyme is hypothesized to be responsible for this biodepolymerization of PVC.

A tandem dehydrochlorination-metathesis process has been reported to depolymerize PVC (Scheme 3C).^82^ Base-mediated elimination first introduces alkene functionalities into the PVC backbone. Applying allyl alcohol as a cosolvent grafts allyl ether groups onto the polymer, which serves as a handle to promote ring-closing metathesis. Applying Grubbs-II catalyst to **13** in the presence of **14** yields a complex mixture of alkenes, with **15** observed as the main low molecular product. A photocatalytic method can oxidize and degrade PVC into acetic acid using a Nb_2_O_5_ photocatalyst (Scheme 3D).^83^ The Nb_2_O_5_ photocatalyst is proposed to facilitate the formation of hydroxyl radicals, which mediate the oxidative C–C bond cleavage of PVC to generate CO_2_. The photoreduction of CO_2_ then generates CO_2_H radicals, which can dimerize and undergo further reduction to form acetic acid. On the other hand, chloride might be oxidized to form Cl_2_ or HOCl. This reaction is also effective in converting PE and polypropylene (PP) into acetic acid.

Despite the commercialization of various PVC recycling methods, significant opportunities remain to enhance the sustainability of these processes, especially considering the challenges associated with handling postconsumer PVC waste. Biodegradation represents an intriguing strategy, as PVC is traditionally considered a nonbiodegradable material. However, the complexity and efficiency of these methods pose substantial challenges in matching the simplicity and scalability of existing gasification and dehydrochlorination processes.

### 3.4 Chemical Recycling of Polypropylene (PP, SPI Code 5)

Polypropylene (PP) is a thermoplastic with the lowest density among commodity plastics.^84^ PP is more rigid and thermally stable compared to other polyolefins like HDPE and LDPE, making it suitable for applications requiring high temperature or chemical resistance, such as food packaging, automotives, construction and textiles.^85^ For example, food containers made from PP are typically microwave and dishwasher safe, unlike those made from PET. Due to its resistance to chemical degradation, PP is also widely used in medical and scientific fields for single-use laboratory consumables like pipettes, syringes, and tubes.

In 2018, approximately 56 million metric tons of PP were produced worldwide, representing the second most produced commodity plastic.^86^ PP is synthesized through chain growth polymerization of propylene using either heterogeneous supported transition metals or homogeneous group 4 metallocene Ziegler–Natta catalysts.^87^ PP can be produced with different stereochemical configurations: isotactic, syndiotactic, and atactic. Commercial Ziegler–Natta catalysts typically make isotactic PP, which means the methyl substituents are arranged on the same side of the polymer backbone. Atactic PP, which has the methyl substituents arranged randomly along the polymer, and syndiotactic PP, which have the methyl substituents arranged on alternating sides of the polymer, exhibit lower melting points compared to isotactic PP and are much less commercially relevant.

PP can be identified with an SPI code of 5 and is less commonly accepted by curbside recycling programs compared to other plastics such as PET and HDPE. Despite its widespread use across numerous industries, the recycling rate for PP in the United States was only 1% in 2017.^88^ Like many thermoplastics, mechanical recycling offers a straightforward method for managing postconsumer PP waste, but it has limitations, such as degradation of the PP backbone through repeated recycling processes. This degradation, which results in lower quality material,^89^ proceeds through hydrogen atom abstraction followed by chain scission and typically occurs at the high temperatures required during the extrusion processes of mechanical recycling. Therefore, incineration and landfilling remain the most common methods for disposing of PP waste.^90^

**Scheme 4:**
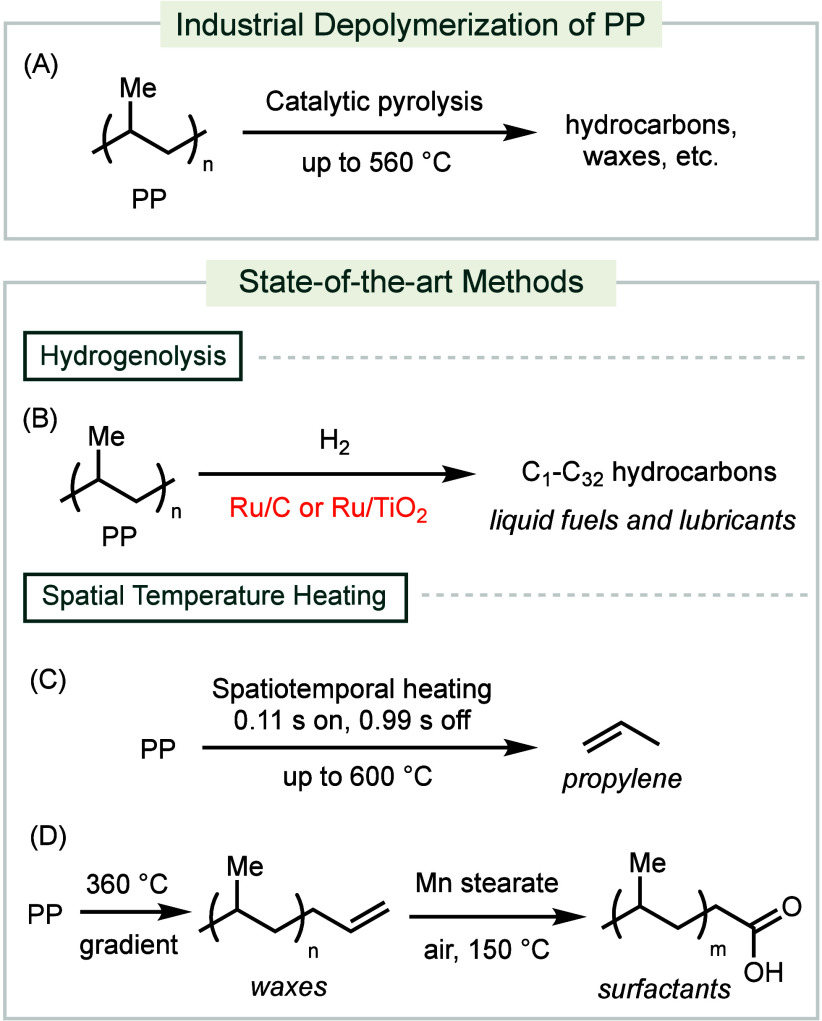
Industrial and Forefront Methods for PP Depolymerization

The chemical recycling of postconsumer PP waste is often treated as a mixture with other polyolefins.^91^ Therefore, many of the pyrolysis technologies discussed above also apply to PP waste, in addition to some commercial pyrolysis processes tailored for PP (Scheme 4A). Encina’s patented process uses a zeolite catalyst to convert PP into feedstock chemicals such as benzene, toluene, xylene, and propylene.^92^ Encina, operating in the United States, plans to build a $1.1 billion facility to implement this recycling technology.^93^ Additionally, Nexus Circular has patented pyrolytic treatments of PP and currently operates a 50 ton/day plant in the United States.^94^

State-of-the-art conditions implement hydrogenolysis to convert PP into mixtures of hydrocarbon products (Scheme 4B). A Ru/C catalyst produces C_5_–C_32_ iso-alkanes at 200–250 °C under 20–50 bar H_2_.^95^ This method has also been demonstrated to be effective with a mixture of PP and PE waste. Additionally, a Ru/TiO_2_ catalyst can convert PP into hydrocarbons that are valuable as lubricants.^96^ The reaction proceeds at 250 °C under 30 bar of H_2_.

Recently, methods that leverage temperature-gradient technologies have been developed to improve selectivity for specific products. A thermochemical depolymerization of PP employs a reactor that induces pyrolysis through electrified spatiotemporal heating (Scheme 4C).^97^ This depolymerization process features a spatial temperature gradient and a temporal heating profile. A porous carbon bilayer is electrically heated, conducting heat to an underlying reactor containing PP. The resulting temperature gradient promotes continuous melting and reaction as the plastic material moves through the carbon bilayer. Pulsing the electrical current allows for a temporal heating profile, enabling precise control over the duration the plastic material is exposed to peak temperatures. This process yields 36% propylene and has also proved effective with PET waste. Moreover, a temperature-gradient reactor has been designed to prevent the complete pyrolysis of PP into small molecules (Scheme 4D).^98^ The waxes produced after thermolysis are subjected to oxidation using a Mn stearate catalyst to produce surfactants. This reactor is also compatible with municipal HDPE and LDPE waste.

### 3.5 Chemical Recycling of Polystyrene (PS, SPI Code 6)

Polystyrene (PS) is a thermoplastic typically characterized by its rigidity and brittleness. It is available in two common forms: solid and foamed.^99^ Solid PS is utilized in various applications across numerous industries, including packaging, consumer goods, and the production of drinking cups and lids. Foamed PS, commercially known as Stryofoam, is a trademark of DuPont and extensively used in both packaging and construction sectors. In 2022, the global production reached approximately 15 million metric tons, making it the fourth most-produced commodity plastic.^100^

PS is predominantly synthesized through bulk free radical polymerization of styrene, employing a radical initiator. Styrene is also frequently copolymerized with butadiene to form high impact polystyrene (HIPS).^101^ Additionally, similar to PP, PS can exhibit various tacticity. In industrial settings, atactic PS is most common, where the stereochemistry α to the phenyl substituents is random.

PS is designated with an SPI code of 6, signaling to consumers that it is not suitable for regular curbside recycling programs and should be segregated from other recyclable plastics. Foamed PS, which can contain up to 95% air, presents particularly formidable recycling challenges due to the logistical and economic difficulties involved in transporting and processing this lightweight waste.^102^ Therefore, only about 1% of postconsumer PS is recycled annually. Like many thermoplastics, PS undergoes significant degradation through mechanical recycling, especially after multiple cycles. Chain scission represents the most prevalent form of degradation during reprocessing, although cross-linking may also occur at higher temperatures. Given these recycling obstacles, the end-of-life management for PS typically involves either landfilling or incineration.

**Scheme 5:**
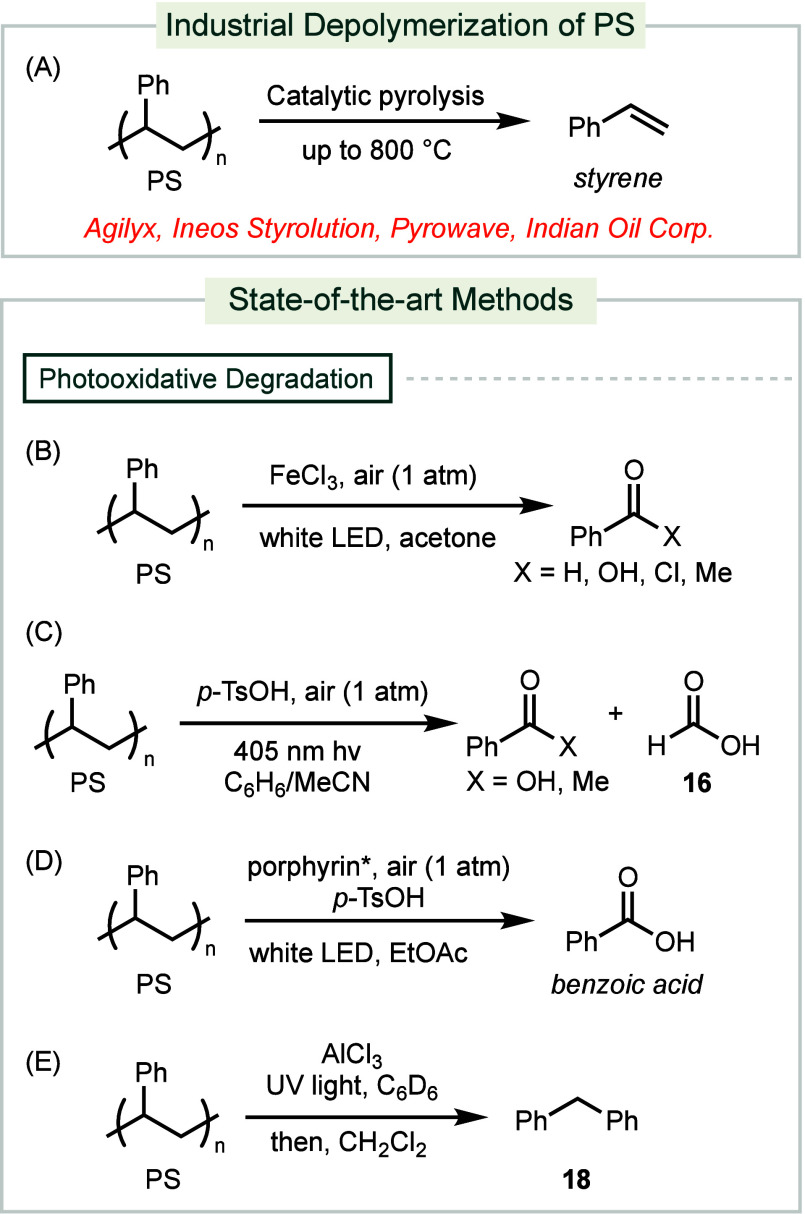
Industrial and Forefront Methods for PS Depolymerization

Several industrial operations are dedicated to recycling PS through purification and reformation processes, including facilities operated by Polystyvert and PolystyreneLoop.^103,104^ Additionally, there are established industrial methods for the chemical recycling of PS, primarily through pyrolysis (Scheme 5A).^105−109^ Agilyx, in partnership with Ineos Styrolution, has announced plans to build a PS recycling plant in the United States with a capacity to process up to 100 tons of waste daily.^110^ Ineos also operates multiple plants in Europe, which collectively convert approximately 15,000 tons of PS into styrene each year.^111^ In Asia, Indian Oil Corp. has developed a catalytic pyrolysis process for the chemical recycling of PS. They are currently advancing plans to establish a commercial plant in India with the capacity to handle 387,000 tons of waste per year.^112^ Analysis of the oil fraction obtained after pyrolysis reveals the formation of styrene in 33% yield, along with monoaromatic products such as toluene, ethylbenzene, and α-methyl styrene. Additionally, poly aromatic products such as stilbene, 1,3-diphenyl propane, and 1,4-diphenyl-1-butene are also present.^113^

Recently, photoexcitation has been explored as a milder and more sustainable alternative to thermal methods for the depolymerization of PS. Irradiation of PS with white LED in the presence of FeCl_3_ as a catalyst at room temperature under ambient atmosphere produces a mixture of oxidized aromatic products with yields up to 23% (Scheme 5B).^114^ The mechanism involves the generation of chlorine radicals that abstract the benzylic hydrogen-atom from PS. The resultant radical is trapped by O_2_ and undergoes β-scission to furnish the cleaved product. Concurrently developed, another photoinduced method employs *p*-TsOH as an acid catalyst and 405 nm light under ambient temperature and atmosphere (Scheme 5C).^115^ Singlet oxygen, generated by blue LED light, abstracts a hydrogen atom from PS. The resulting radical is subsequently trapped by O_2_, leading to β-scission and the production of a mixture of oxidized aromatic products along with formic acid **16**.

Recently, a photoinduced oxidative reaction uses a porphyrin derivative as the photocatalyst to generate singlet oxygen through UV light irradiation, yielding benzoic acid up to 71% (Scheme 5D).^116^ Additionally, a light-induced depolymerization of PS, utilizing catalytic AlCl_3_ under ambient temperature and pressure, produces benzene as the major product (Scheme 5E).^117^ With the addition of CH_2_Cl_2_, diphenylmethane **18** is formed in 87% yield via a Friedel–Crafts reaction. This process has been demonstrated to be scalable to a ton-scale batch reactor.

Although promising methods for PS chemical recycling have been developed in both industry and academia, significant challenges remain in the logistics and cost to collect and transport foamed PS. Currently, it is more cost-efficient to simply landfill or incinerate this type of postconsumer waste. Thus, there is still a need for more cost-effective chemical recycling processes.

### 3.6 Chemical Recycling of Other Commodity Polymers (SPI Code 7)

SPI code 7 includes a wide range of common plastics that lack standardized recycling protocols and are not produced on the same scale as the first six commodity plastics. Notable examples of plastics in this miscellaneous category include poly(methyl methacrylate) (PMMA), polycarbonate (PC), polyamide (PA, or Nylon), and polysiloxane.

#### 3.6.1 Chemical Recycling of Poly(methyl methacrylate) (PMMA)

Poly(methyl methacrylate) (PMMA), also known as Plexiglas, is a transparent thermoplastic often used as a cheaper, lightweight, and shatter-proof alternative to glass. Annually, approximately 3.9 million metric tons of PMMA are produced globally through the free radical polymerization of methyl methacrylate (MMA).^118,119^ Despite its broad applications in consumer goods, electronics, construction, and automobiles, only about 10% of PMMA waste is recycled each year. Landfilling and incineration remain the predominant end-of-life treatment for postconsumer PMMA waste.

**Scheme 6:**
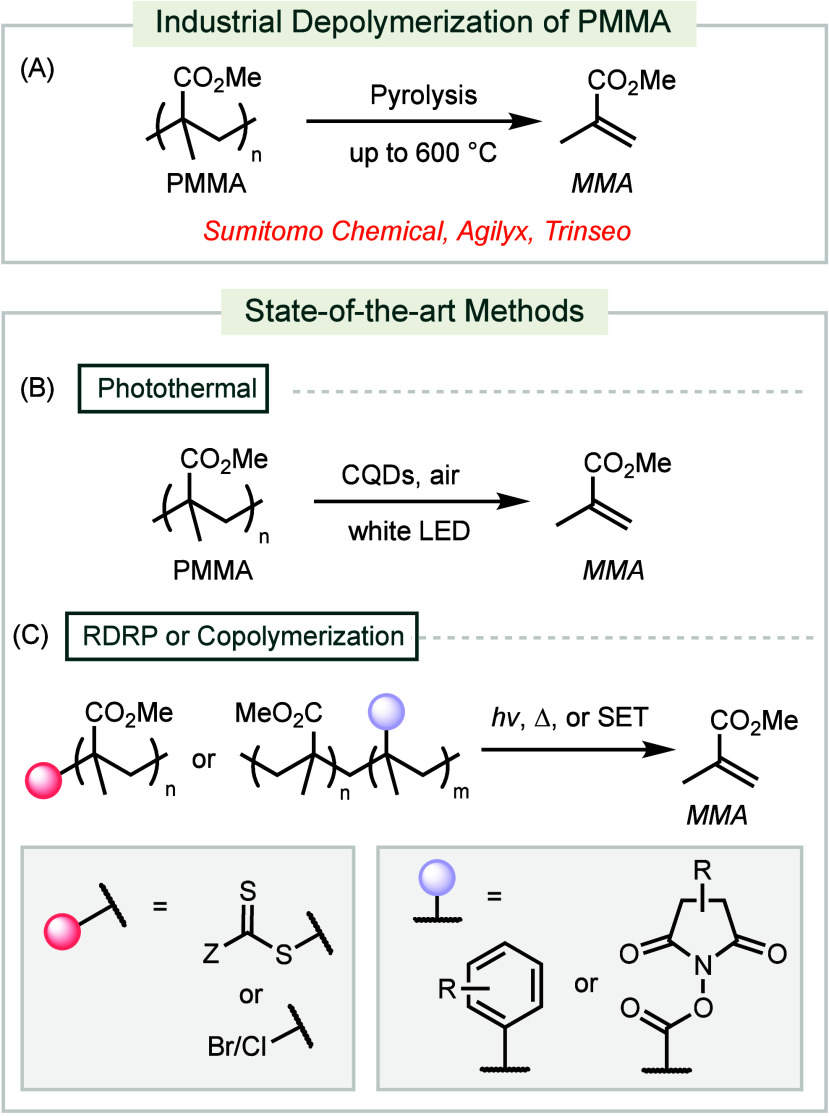
Industrial and Forefront Methods for Depolymerization of PMMA

Current industrial technologies implement thermal gasification and pyrolysis to convert PMMA waste into MMA (Scheme 6A).^120−124^ The pyrolytic depolymerization mechanism is proposed to proceed through the homolytic scission of main-chain C–C bonds, followed by radical depropagation.^125^ In 2021, Agilyx, in collaboration with Mitsubishi Chemical Corp., successfully operated a pilot-scale facility for the pyrolytic depolymerization of PMMA, with plans to further commercialize the process.^126^ In Europe, a collaboration between Trinseo and Japan Steel Works leads efforts in the chemical recycling of PMMA, with a depolymerization plant set to be commissioned in Italy in 2024.^127^ Additionally, in Asia, Sumitomo Chemical has partnered with Japan Steel Works to build a pilot facility that will convert PMMA waste into MMA.^128^

A recent advancement in chemical depolymerization directly converts PMMA to MMA under photothermal conditions (Scheme 6B).^129^ This method employs carbon quantum dots (CQDs) as a photothermal agent. Upon irradiation, a temperature gradient is established near the CQD surface, inducing the thermal cleavage of proximal C–C bonds. This process has also been successfully applied to other polymer classes, including poly(α-methylstyrene) (PAMS) and PS.

Additionally, modern depolymerization methods for PMMA leverage labile end-groups from reversible-deactivation radical polymerization (RDRP) or comonomers from bulk polymerization to reduce the initial decomposition temperature (Scheme 6C).^130−138^ These labile groups are typically activated by light, heat, or single-electron transfer (SET). While these approaches are efficient, mild, and yield high-quality MMA, they are not applicable to existing PMMA waste.

#### 3.6.2 Chemical Recycling of Polycarbonate (PC)

Polycarbonate (PC) refers to a group of transparent thermoplastics often compared to PMMA but noted for their greater tensile strength and higher thermal stability.^139^ The most commonly used type is poly(bisphenol A carbonate) (BPA-PC), synthesized through a base-mediated addition of bisphenol A (BPA) to phosgene. Alternatively, nonphosgene methods using CO_2_ as a raw material can avoid using highly toxic phosgene.^140^ PCs are used across various industrial sectors, including consumer products, electrical, construction, and automobiles, and are also employed in specialized applications such as bulletproof glass.^141^ More recently, due to the potential adverse health effects of BPA, the FDA, EU, and Canada have prohibited its use in food packaging and infant bottles. As a result, other compounds, including benzoic acid, bisphenol A diglycidyl ether (BADGE), and butylated hydroxytoluene (BHT), have been explored as nontoxic alternatives.^142^ In 2016, global production of PCs totaled approximately 4.4 million metric tons.^143^

**Scheme 7:**
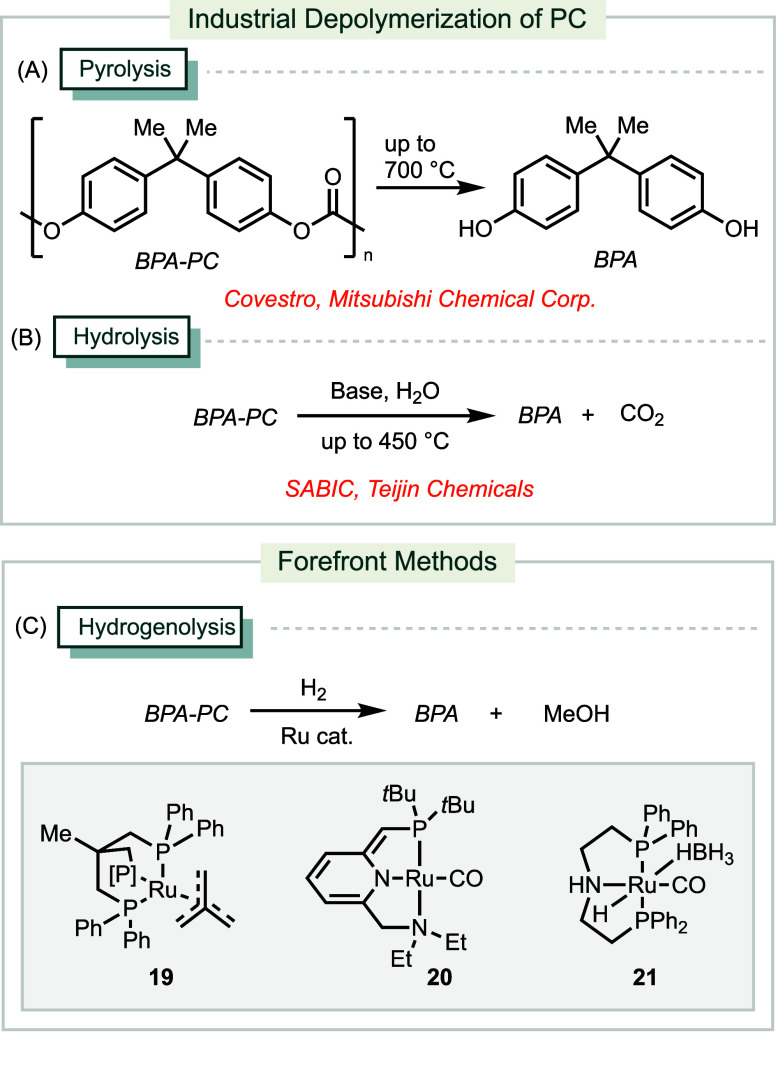
Industrial and Forefront Methods for Depolymerization of PC

Similar to other plastics, the mechanical recycling of PCs results in materials with diminished properties, such as reduced impact resistance, after repeated recycling processes.^144^ Although pyrolysis of PCs yields complex mixtures of aromatic products, there are industrial methods that recover bisphenol A (BPA) through pyrolytic treatments (Scheme 7A).^145−147^ Covestro operates a pilot-scale pyrolysis plant in Germany that converts PC back into its monomers.^148^ Mitsubshi Chemical Corp. has also announced plans to build a pilot facility, with future expansions projected to reach a capacity of 10,000 tons per year.^149^ Hydrolysis of the PC backbone offers a milder and more selective method for recovering BPA compared to pyrolysis (Scheme 7A). This process typically involves the use of a metal hydroxide in aqueous solutions at elevated temperatures, yielding BPA and CO_2_. These conditions have been patented by companies such as SABIC and Teijin Chemicals.^150−152^ Modern methods for the depolymerization of PC employ catalysts to improve the efficiency and selectivity of hydrolysis or pyrolysis.^153^

Hydrogenolysis presents a promising alternative to hydrolysis and pyrolysis for the depolymerization of PC, primarily producing BPA and methanol (Scheme 7C). Applying 0.5 mol % of Ru catalyst **19** at 140 °C under 100 bar of H_2_, this method can convert a PC compact disc (CD) into BPA with a 73% isolated yield.^154^ More recently, employing 5 mol % of Milstein’s pincer Ru catalyst **20** under the same conditions has been shown to achieve a quantitative yield of BPA.^155^ Additionally, a reaction using 0.5 mol % of Ru catalyst **21** at a lower temperature of 80 °C under 45 bar of H_2_ has also yield BPA quantitatively.^156^

#### 3.6.3 Chemical Recycling of Nylon

Poly(amide), commonly known as Nylon, is a class of thermoplastics known for being soft with high elongation and abrasion resistance.^157^ Nylon is typically processed into fibers, which are widely used in textiles for clothing, carpets, and other consumer goods. Nylon is also molded into resins and used in the automobile industry. Nylon can be synthesized through polycondensation between diamines and dicarboxylic acids, or via the hydrolytic polymerization of lactams.^158,159^ The chain length of the monomers determines the nomenclature of the resulting polymer; for example, Nylon-6,6 is synthesized from adipic acid and hexamethylenediamine, both containing 6 carbon atoms, whereas Nylon-1,6 is formed from formaldehyde and adiponitrile. In 2019, 5.6 million tons of Nylon were produced, of which only 2% was manufactured using recycled materials.^160^

**Scheme 8:**
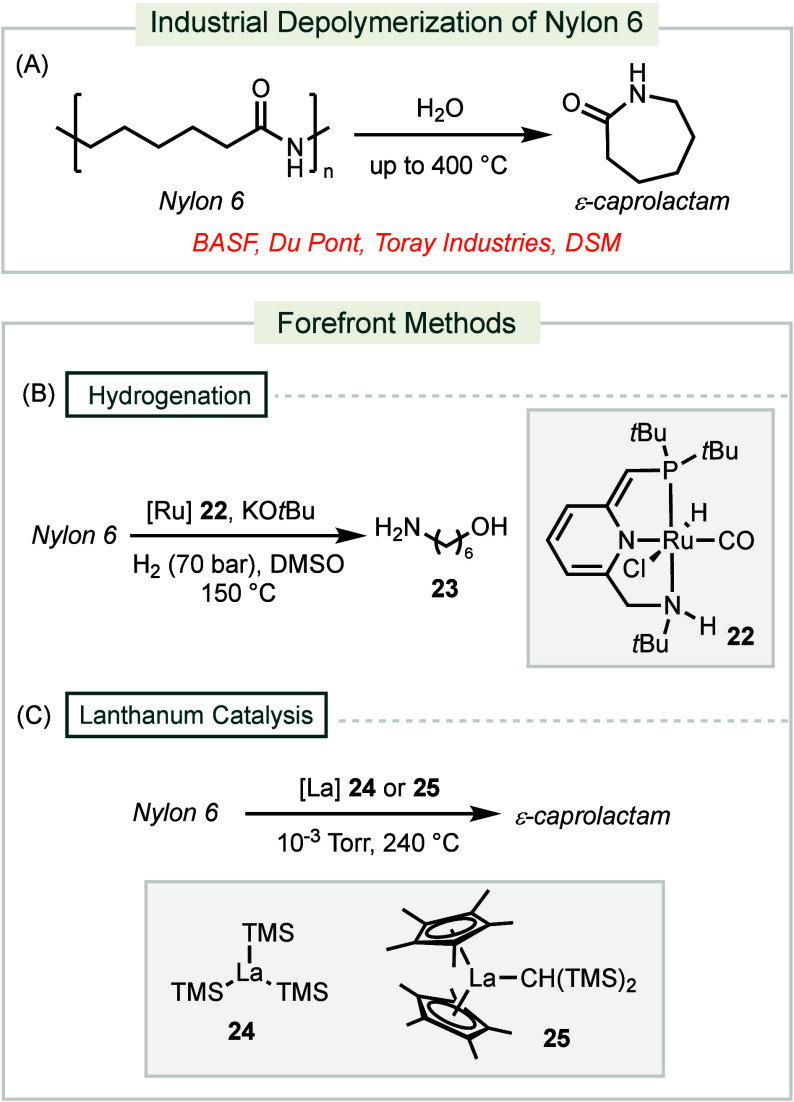
Industrial and Forefront Methods for Depolymerization of Nylon 6

Mechanical recycling of Nylon can lead to hydrolysis of the polyamide backbone at elevated extrusion temperatures, resulting in a lower quality material.^147^ Due to the susceptibility of Nylon to hydrolyze, many industrial developments leverage this reactivity to recover monomers, a process that can occur both with and without acid or base catalysts (Scheme 8A).^161−167^ Notably, conditions reported by companies such as BASF, Du Pont, and Toray Industries focus on Nylon-6, with ε-caprolactam being recovered as the monomeric product. In 2023, Toray Industries announced a partnership with Honda Motor Co. to develop a 500 t/year pilot facility to recover ε-caprolactam from Nylon-6 waste.^168^ This technology utilizes supercritical water at elevated temperatures and operates catalyst-free. Asahi Kasei also has announced a partnership with Microwave Chemical to implement microwave technology for the depolymerization of Nylon-6,6 to recover adipic acid and hexamethylenediamine, which have plans to build a small-scale operation in 2024.^169^

Advanced depolymerization techniques employ transition-metal catalysts to process Nylon under conditions that are milder and more selective than typical industrial methods. Hydrogenation with a pincer ruthenium catalyst **22** at 150 °C under 70 bar H_2_ allows for the conversion of Nylon-6 into amino alcohol **23** with a 24% yield (Scheme 8B).^170^ The proposed mechanism involves hydride transfer from a ruthenium dihydride intermediate to the polyamide, resulting in the formation of a hemiaminal. Under basic conditions, the hemiaminal undergoes C–N bond cleavage, yielding an amine and an aldehyde; the latter is then reduced to an alcohol. This reaction has proven effective across a wide spectrum of Nylon polymers.

The application of 5 mol % of lanthanide catalyst **24** can depolymerize Nylon-6 into ε-caprolactam with a 90% yield at 240 °C under a static vacuum of 10^–3^ Torr (Scheme 8C).^171^ This lanthanide catalyst acts as a Lewis-acid, facilitating the intramolecular cyclization of the polymer backbone to release ε-caprolactam. Reactivity has been further improved by using 1 mol % of lanthanide catalyst **25** at the same temperature, achieving 99% yield of ε-caprolactam. The bulky bis(cyclopentadienyl) (Cp*) ligands help minimize catalyst deactivation previously observed with **24**.^172^ This method is effective across a broad range of postconsumer Nylon-6 waste materials.

#### 3.6.4 Chemical Recycling of Polysiloxane

Polysiloxane is a class of thermoplastics characterized by a Si–O backbone with alkyl or aryl substituents. Depending on the polymer chain length, end groups, and the addition of fillers or additives, these materials can exist as either oils or rubber-like substances.^173^ Polydimethylsiloxane (PDMS), also known as dimethicone, is one of the most common silicone polymers. PDMS is used in a broad range of applications, from silicone oil and lubricants to silicone rubber and cosmetics.^174^ The industrial synthesis of PDMS involves the polymerization of dimethylchlorosilane with water, which releases HCl as a stoichiometric byproduct. In 2013, an estimated 2.1 million tons of polysiloxanes were produced worldwide.^175^

Depolymerization of PDMS typically occurs through Si–O bond cleavage, which can be mediated by catalytic or stoichiometric acids and bases.^176−179^ Initially, hydrolytic cleavage of a main chain Si–O bond forms a silanolate, which undergoes intramolecular σ-bond metathesis, known as “backbiting”, releasing cyclic siloxanes as products. This mechanism can also be initiated by nucleophiles and electrophiles or by heating above 350 °C in the presence of catalysts.^163,180^ The thermodynamically favored product is hexamethylcyclotrisiloxane (D_3_); however, octamethylcyclotrisiloxane (D_4_), decamethylcyclotrisiloxane (D_5_), and dodecamethylcyclotrisiloxane (D_6_) can also be formed at lower temperatures under specific conditions. At temperatures above 500 °C, homolytic scission of Si–C side chain bonds can occur, leading to the generation of methane radicals and cross-linking of PDMS chains.^181^

**Scheme 9:**
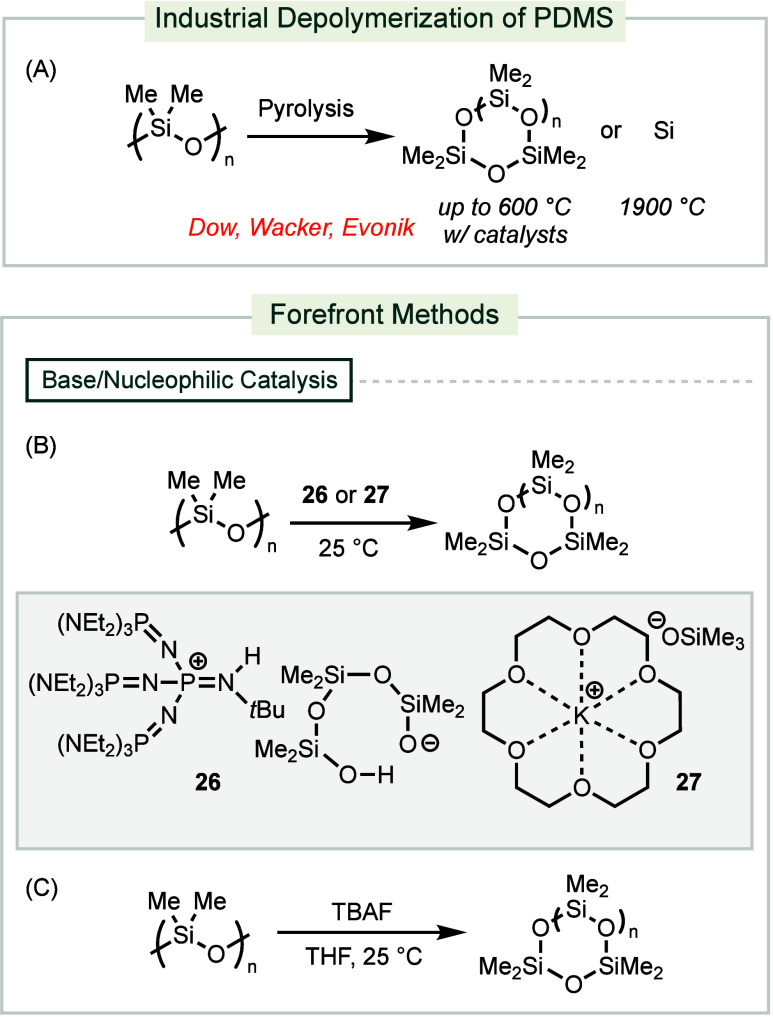
Industrial and Forefront Methods for Depolymerization of PDMS

Industrial processes utilize both catalytic and noncatalytic pyrolysis to depolymerize PDMS into cyclic siloxanes (Scheme 9A).^182^ Dow Corning has utilized various heterogeneous Lewis acids to convert PDMS into a mixture of cyclic siloxanes at 350 °C.^183^ Additionally, Dow has announced a partnership with Circusil to build a silicone recycling plant in the United States, scheduled to commence operations in 2024.^184^ Similarly, Evonik has developed processes using Brønsted acids, which also generate cyclic siloxanes as depolymerization products at temperatures up to 600 °C.^185,186^ Wacker Chemie has developed noncatalytic methods where PDMS waste is transformed into metallurgic silicon at temperatures reaching 1900 °C.^187^

State-of-the-art conditions aim to enhance base-mediated and nucleophilic mechanisms for PDMS recycling. A phosphazenium silanolate catalyst **26** facilitated the depolymerization of PDMS with a 0.1 mol % loading and at ambient temperature to afford a mixture of cyclic siloxanes in 82% yield (Scheme 9B).^188^ A crown ether-ligated silanolate catalyst **27** promoted the depolymerization of PDMS to cyclic siloxanes with up to 99% yield at 140 °C.^189^ The reaction proceeds without the need for a solvent, and products can be easily recovered through vacuum distillation. Leveraging the propensity for Si–F bond formation, 0.5 mol % of TBAF at ambient temperature depolymerizes PDMS to a mixture of cyclic siloxanes (Scheme 9C).^190^ The depolymerization process is initiated by the addition of fluorine to a silicon center within the polymer backbone. After the cleavage of the Si–O bond, a silanolate is formed, which can undergo backbiting to release cyclic siloxanes.

#### 3.6.5 Chemical Recycling of Epoxy Resin

Epoxy resin is a class of thermoset plastics characterized by extensive cross-linking.^191^ Due to its favorable properties in electrical insulation, solvent resistance, and mechanical strength, epoxy resins have many applications, including packaging, adhesives, and reinforced composites.^192^ The synthesis of epoxy resins typically involves the condensation of an epoxide monomer with two or more oxirane groups with a curing agent, which can include both aliphatic and aromatic amines, amides, phenols, and acids. Bisphenol A (BPA) is the most used curing agent. In 2022, the global production of epoxy resins exceeded 6 million tons.^193^

**Scheme 10:**
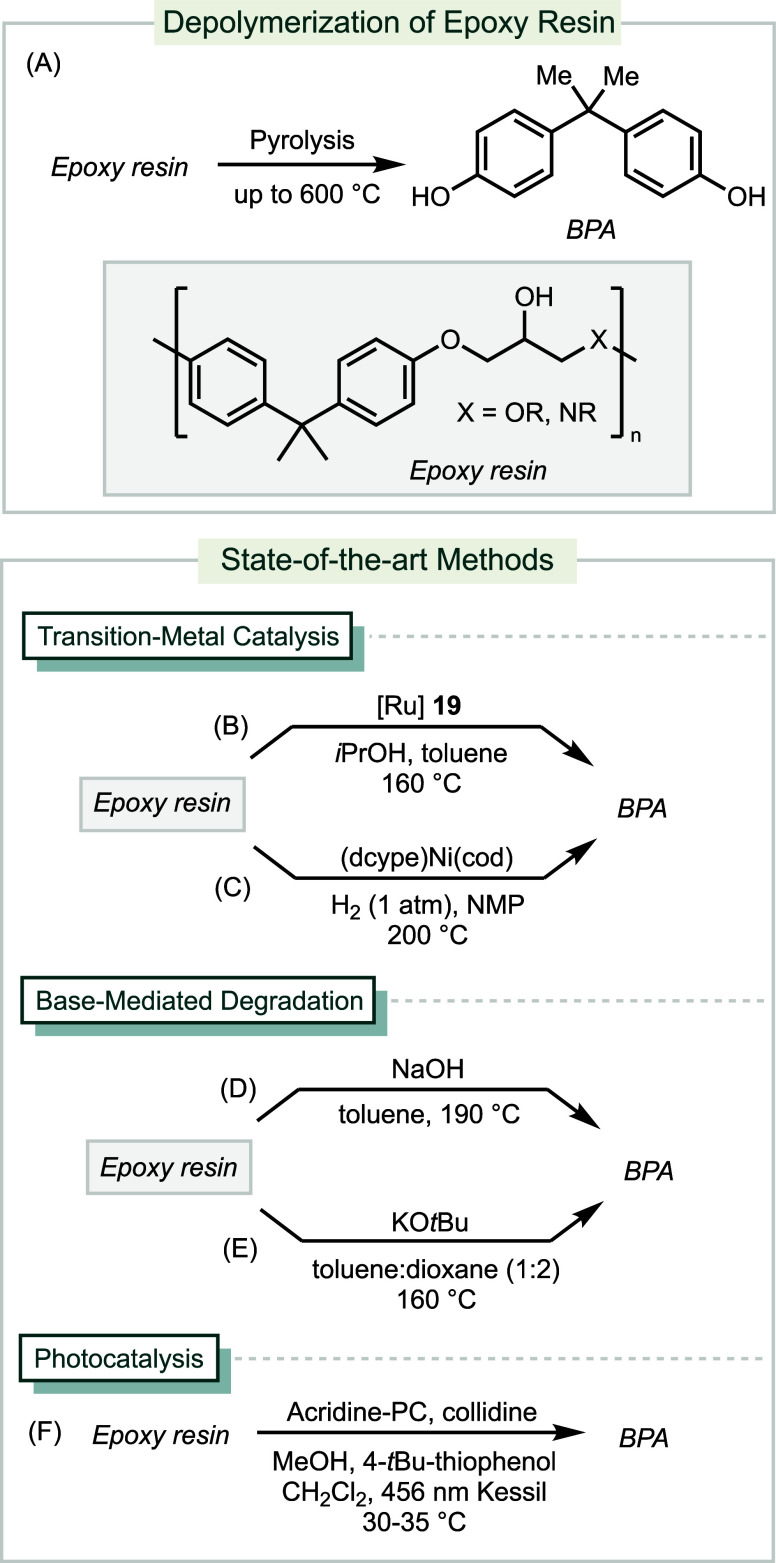
Methods for Depolymerization of Epoxy Resins

Pyrolysis of epoxy resins presents a straightforward method to convert postconsumer waste into monomers, but necessitates high temperature conditions, which are not sustainable (Scheme 10A).^194^ State-of-the-art methods aim to offer milder conditions by applying transition-metal catalysis or base-mediated degradation. The use of ruthenium catalyst **19** has been shown to deconstruct epoxy resins into BPA (Scheme 10B).^195^ The reaction proceeds through dehydrogenation of the secondary alcohol, followed by oxidative addition of Ru into the adjacent PhO–C bond. The reaction is compatible with several types of commercial epoxy resins, recovering up to 81% yield of BPA. Nickel-catalyzed hydrogenolysis of epoxy resins produces BPA in 66% isolated yield from a diamine-cured epoxy resin (Scheme 10C).^196^ NaOH or KO*t*Bu can mediate the degradations of epoxy resins to yield BPA in up to 81% and 71% yield, respectively (Scheme 10D and 10E).^197,198^ Lastly, a photocatalytic method with an acridine photocatalyst converts thiol-cured epoxy resins into BPA (Scheme 10F).^199^ The reaction operates through a proton-coupled electron transfer (PCET) mechanism, which promotes C–C cleavage through β-scission.

### 3.7 Forefront Methods Targeting Multiple SPI Codes

Currently, technologies for sorting postconsumer plastic waste are time-consuming, expensive, or have inherent limitations. A typical protocol applies a combination of techniques, including manual sorting, sink-float, and infrared (IR) spectroscopy.^200^ Manual sorting, which relies on recognizing SPI codes, is inefficient and cost-intensive. Sink-float methods, which exploit differences in the densities of each type of plastic, can be expensive due to the need for dedicated washing, drying, and wastewater treatments.^201^ Sorting processes based on IR spectroscopy use an IR detector to identify different types of plastics. Although these IR techniques are typically automated, they have limitations, such as difficulty analyzing colored or contaminated materials, which can interfere with IR detection and impede accurate identification.^200^

Conditions that convert various types of plastics into valuable chemicals or raw materials could eliminate the need for rigorous sorting of plastic waste. Several examples demonstrate compatibility with different materials, including those capable of recycling both HDPE and LDPE. For example, the oxidative degradation of PE could also be applied to PS and PET.^55^ Similarly, the photocatalytic conditions were shown to convert PE and PP, in addition to PVC, into acetic acid.^76^ Additionally, the photothermal conditions to depolymerize PMMA are also effective in depolymerizing PS and polylactic acid (PLA) into monomers.^121^

**Scheme 11:**
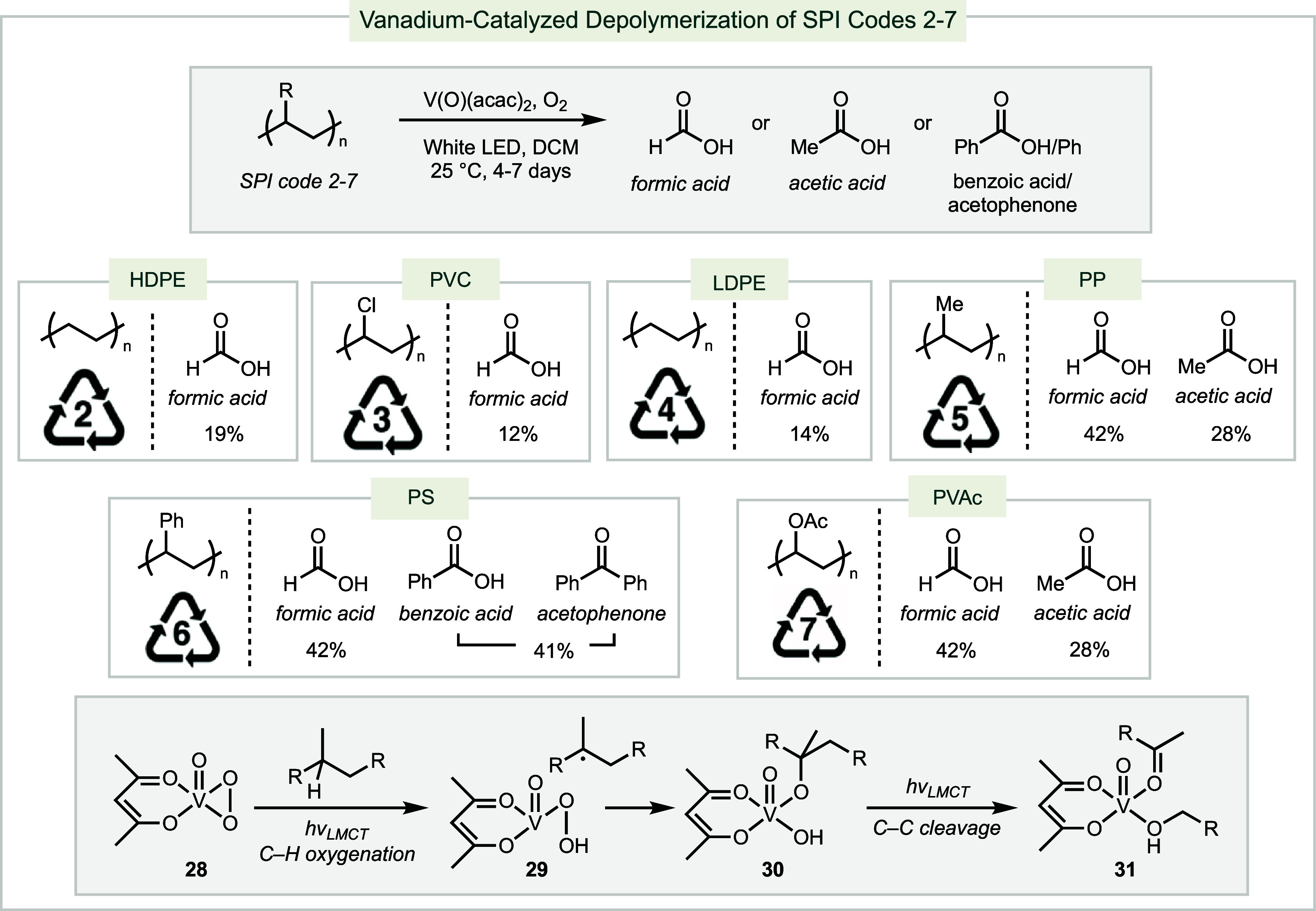
Vanadium-photocatalyzed Depolymerization of SPI Codes 2-7

More recently, a photocatalytic conditions employing commercially available vanadium catalyst V(O)(acac)_2_ have been reported to convert SPI codes 2–7 into valuable chemicals such as formic acid, benzoic acid, and acetic acid (Scheme 11).^202^ The vanadium peroxide catalyst **28**, generated from V(O)(acac)_2_ and O_2_, can be photoexcited through ligand-to-metal charge transfer (LMCT). The triplet excited state of the catalyst conducts H-atom abstraction to generate a radical within the polymer, which is trapped by V(IV) **29** to afford intermediate **30**. Photoexcitation of **30** triggers LMCT to access its triplet excited state, which facilitates C–C bond cleavage through alkyl group transfer to generate **31**. This process has been shown to be compatible with HDPE, PVC, LDPE, PP, PS, and SPI code 7 plastics such as polyvinyl acetate (PVAc) and poly(ethylene-vinyl acetate) (PEVA).

## 4 Conclusion

Chemical recycling of plastics provides access to valuable chemicals and high-quality raw materials from postconsumer waste, which can be reprocessed into materials with properties comparable to those of virgin materials. This cyclical process of polymerization and depolymerization promotes a circular economy with favorable economic and environmental implications. Pyrolysis and methods involving inexpensive commodity chemicals are standard practice for chemical recycling of plastics in the industry due to their simplicity and cost-effectiveness. However, these methods often require high temperatures and/or pressures, making them energy-intensive and less sustainable for chemical recycling. Thus, there is a significant challenge and opportunity to develop more efficient, cheaper, and milder methods.

Two approaches can improve plastic waste management: (1) developing improved catalytic conditions for chemical recycling, and (2) creating new plastics more amenable to large-scale chemical recycling. The former approach relies on several considerations for catalyst and condition development, including low operating costs, stability against air, moisture, and contaminants, compatibility with heterogeneous systems, and efficient conversion to monomer.^203^ Ideally, these conditions would also be solvent-free, rapid, function at modest temperatures. To date, most advancements have involved adapting known reactions and catalysts from small molecules to polymers. However, the physical properties of plastics, including their poor solubility, have posed restrictions on many catalytic systems. Additionally, achieving selectivity in polymer remains a challenge; for example, reactions involving radical intermediates frequently lead to cross-linking. Looking ahead, developing new catalysts especially tailored for polymers to facilitate reaction occurring at the interface of the plastic and solution phases could have a significant impact.

Alternatively, novel plastic materials that retain favorable mechanical and thermal properties could be depolymerized under conditions or stimuli that are milder and more cost-effective than current industrial standards.^204^ While this approach offers a promising solution for the future, it does not address the issue of existing waste material and may encounter significant barriers to adoption within the commodity production industry.

## References

[ref1] Geyer R.; Jambeck J. R.; Law K. L. Production, use, and fate of all plastics ever made. Sci. Adv. 2017, 3, e1700782 10.1126/sciadv.1700782. 28776036 PMC5517107

[ref2] IEA The Future of Petrochemicals; IEA, Paris, France, 2018. https://www.iea.org/reports/the-future-of-petrochemicals.

[ref3] OECD. Plastic pollution is growing relentlessly as waste management and recycling falls short, says OECD. Organisation for Economic Co-operation and Development, 2022.https://www.oecd.org/en/about/news/press-releases/2022/02/plastic-pollution-is-growing-relentlessly-as-waste-management-and-recycling-fall-short.html (accessed 2024-03-02).

[ref4] Hopewell J.; Dvorak R.; Kosior E. Plastics recycling: challenges and opportunities. Philos. Trans. R Soc. London B Biol. Sci. 2009, 364, 2115–26. 10.1098/rstb.2008.0311. 19528059 PMC2873020

[ref5] Coates G. W.; Getzler Y. D. Y. L. Chemical recycling to monomer for an ideal, circular polymer economy. Nat. Rev. Mater. 2020, 5, 501–516. 10.1038/s41578-020-0190-4.

[ref6] Lohmann V.; Jones G. R.; Truong N. P.; Anastasaki A. The thermodynamics and kinetics of depolymerization: what makes vinyl monomer regeneration feasible?. Chem. Sci. 2024, 15, 832–853. 10.1039/D3SC05143A. 38239674 PMC10793647

[ref7] Rahman M.; Weale K. E. The effect of pressure on ceiling-temperature in the polymerization of tetrahydrofuran. Polymer 1970, 11, 122–124. 10.1016/0032-3861(70)90082-0.

[ref8] Ivin K. J. Thermodynamics of addition polymerization. J. Polym. Sci. A Polym. Chem. 2000, 38, 2137–2146. 10.1002/(SICI)1099-0518(20000615)38:12<2137::AID-POLA20>3.0.CO;2-D.

[ref9] Martinez M. R.; Krys P.; Sheiko S. S.; Matyjaszewski K. Poor Solvents Improve Yield of Grafting-Through Radical Polymerization of OEO19MA. ACS Macro Lett. 2020, 9, 674–679. 10.1021/acsmacrolett.0c00245. 35648572

[ref10] Otsu T.; Yamada B.; Mori T.; Inoue M. Ceiling temperatures in radical polymerizations of N-phenyl and N-n-butyl methacrylamide. J. Polym. Sci., Polym. Lett. 1976, 14, 283–285. 10.1002/pol.1976.130140506.

[ref11] Diaz-Silvarrey L. S.; Zhang K.; Phan A. N. Monomer recovery through advanced pyrolysis of waste high density polyethylene (HDPE). Green Chem. 2018, 20, 1813–1823. 10.1039/C7GC03662K.

[ref12] Yu J.; Sun L.; Ma C.; Qiao Y.; Yao H. Thermal degradation of PVC: A review. Waste Manage. 2016, 48, 300–314. 10.1016/j.wasman.2015.11.041. 26687228

[ref13] Wesolowski R. A.; Wesolowski A. P.; Petrova R. S. Polymer Identification Code. In The World of Materials; Springer International Publishing, 2020; pp 117–123.

[ref14] Bouhlel Z., Köpke J.; Mina M.; Smakhtin V. Global Bottled Water Industry: A Review of Impacts and Trends United Nations; University Institute for Water, Environment and Health: Hamilton, Canada, 2023.

[ref15] Smith R. L.; Takkellapati S.; Riegerix R. C. Recycling of Plastics in the United States: Plastic Material Flows and Polyethylene Terephthalate (PET) Recycling Processes. ACS Sus. Chem. Eng. 2022, 10, 2084–2096. 10.1021/acssuschemeng.1c06845. PMC9004285 35425669

[ref16] Benyathiar P.; Kumar P.; Carpenter G.; Brace J.; Mishra D. K. Polyethylene Terephthalate (PET) Bottle-to-Bottle Recycling for the Beverage Industry: A Review. Polymers (Basel) 2022, 14 (12), 2366 10.3390/polym14122366. 35745942 PMC9231234

[ref17] Köpnick H.; Schmidt M.; Brügging W.; Rüter J.; Kaminsky W. Polyesters. In Ullmann’s Encyclopedia of Industrial Chemistry; Wiley-VCH Verlag GmbH & Co., 2000; pp 623–646.

[ref18] McNeeley A.; Liu Y. A. Assessment of PET Depolymerization Processes for Circular Economy. 1. Thermodynamics, Chemistry, Purification, and Process Design. Process Design. Ind. Eng. Chem. Res. 2024, 63, 3355–3399. 10.1021/acs.iecr.3c04000.

[ref19] Barnard E.; Rubio Arias J. J.; Thielemans W. Chemolytic depolymerisation of PET: a review. Green Chem. 2021, 23, 3765–3789. 10.1039/D1GC00887K.

[ref20] Biermann L.; Brepohl E.; Eichert C.; Paschetag M.; Watts M.; Scholl S. Development of a continuous PET depolymerization process as a basis for a back-to-monomer recycling method. Green Process. Synth. 2021, 10, 361–373. 10.1515/gps-2021-0036.

[ref21] Parravicini M.; Crippa M.; Bertele M. V. Method and apparatus for the recycling of polymeric materials via depolymerization process. WO 2013014650 A1, 2012.

[ref22] Crippa M. Method for the recycling of a textile waste comprising a cellulosic component and a polyester component. WO 2022195433 A1, 2022.

[ref23] Sramek J.; Trzewiczek M.; Travnicek K. Method of obtaining terephthalic acid from waste polyethylene terephthalate. WO 2019174656 A1, 2019.

[ref24] Anderson S.; Ireland C.; Smit B.; Stylianou K. Degradation of plastic materials into terephthalic acid (TPA), ethylene glycol and/or other monomers that form the plastic materials. WO 2020173961 A1, 2020.

[ref25] Tournier V.; Topham C. M.; Gilles A.; David B.; Folgoas C.; Moya-Leclair E.; Kamionka E.; Desrousseaux M. L.; Texier H.; Gavalda S.; Cot M.; Guémard E.; Dalibey M.; Nomme J.; Cioci G.; Barbe S.; Chateau M.; André I.; Duquesne S.; Marty A. An engineered PET depolymerase to break down and recycle plastic bottles. Nature 2020, 580, 216–219. 10.1038/s41586-020-2149-4. 32269349

[ref26] Carbios launches industrial demonstration plant for its unique enzymatic recycling technology; Carbios: Clermont-Ferrand. Carbios, 2021. https://www.carbios.com/en/carbios-annonce-le-demarrage-de-son-demonstrateur-industriel-exploitant-sa-technologie-unique-de-recyclage-enzymatique-c-zyme/(accessed 2024-02-13).

[ref27] Castillo S. I.-M. R.; Berkum S. V.; Philippi V. G. A.; Wolters J. R. Improved catalyst complex and method of degradation of a polymer material. WO 2017111602 A1, 2017.

[ref28] Fufachev E. V.; Wolters A. T.; De Haan A. B.; Wolters J. R. Method and reactor system for depolymerizing a terephthalate-polymer into reusable raw material. WO 2022271013 A1, 2022.

[ref29] Atkins M.; Curry N.; Evans S. Polymer recycling. WO 2022171874 A1, 2021.

[ref30] Brons M.; Ruesink M. A.; Albert J.; Kunst F.; Hoffard J. G. N.; Goor L. V. H.; Lange M. D.; Schmidt B. W.; Jager J. A method to enable recycling of polyester waste material and a system for applying the method. WO 2022003084 A1, 2021.

[ref31] Parrott M.; Luft J. C.; Shuping D. B.; Mattiace M. D. Process and system for depolymerizing waste plastic. Process and system for depolymerizing waste plastic. WO 2021151071 A1, 2021.

[ref32] van Berkum S.; Philippi V.; Marcel A. A.; de Groot R.; Hooghoudt T. Improved reusable capture complex. WO 2016105198 A1, 2016.

[ref33] Ioniqa takes first 10 kiloton PET upcycling factory into operation. Ioniqa, 2019. https://ioniqa.com/ioniqa-takes-first-10-kiloton-pet-upcycling-factory-into-operation (accessed 2024-02-13).

[ref34] Essaddam A.; Essaddam F. Terephthalic acid esters formation. US 20230125080 A1, 2023.

[ref35] Essaddam A.; Essaddam F. Process for the depolymerization of polyethylene terephthalate (pet). WO 2020188359 A1, 2020.

[ref36] Essaddam A.; Essaddam F. Terephthalic acid esters formation. WO 2019051597 A1, 2019.

[ref37] Essaddam A.; Essaddam F. Terephthalic acid esters formation. WO 2020002999 A2, 2020.

[ref38] Medimagh R. Improved method for recycling pet by alcoholysis. WO 2022112715 A1, 2022.

[ref39] Jackson A.-M. S.; Keever T. W.; Ekart M. P. Production of virgin-quality pet and copolyester raw materials from polyester carpet fibers. WO 2023059579 A1, 2023.

[ref40] Eastman and Governor Lee announce world-scale plastic-to-plastic molecular recycling facility to be built in Kingsport, Tenn.. Eastman Chemical Co, 2021. https://www.eastman.com/en/media-center/news-stories/2021/governor-lee-announce-plastic-recycling-facility (accessed 2024-02-13).

[ref41] Kim N.-K.; Lee S.-H.; Park H.-D. Current biotechnologies on depolymerization of polyethylene terephthalate (PET) and repolymerization of reclaimed monomers from PET for bio-upcycling: A critical review. Bioresour. Technol. 2022, 363, 127931 10.1016/j.biortech.2022.127931. 36100185

[ref42] Cao F.; Wang L.; Zheng R.; Guo L.; Chen Y.; Qian X. Research and progress of chemical depolymerization of waste PET and high-value application of its depolymerization products. RSC Adv. 2022, 12, 31564–31576. 10.1039/D2RA06499E. 36380916 PMC9632252

[ref43] Amalia L.; Chang C.-Y.; Wang S. S. S.; Yeh Y.-C.; Tsai S.-L. Recent advances in the biological depolymerization and upcycling of polyethylene terephthalate. Curr. Opin. Biotechnol. 2024, 85, 103053 10.1016/j.copbio.2023.103053. 38128200

[ref44] Shi L.; Zhu L. Recent Advances and Challenges in Enzymatic Depolymerization and Recycling of PET Wastes. ChemBioChem. 2024, 25, e202300578 10.1002/cbic.202300578. 37960968

[ref45] Cecchin G.; Morini G.; Piemontesi F. Ziegler-Natta Catalysts. In Kirk-Othmer Encyclopedia of Chemical Technology; John Wiley & Sons, Inc., 2003; pp 502–554.

[ref46] Malpass D. B., Commercially Available Metal Alkyls and Their Use in Polyolefin Catalysts. In Handbook of Transition Metal Polymerization Catalysts; John Wiley & Sons, Inc., 2010; pp 1–28.

[ref47] Okamura T.-a. Polyethylene (PE; Low Density and High Density). In Encyclopedia of Polymeric Nanomaterials; Kobayashi S., Müllen K., Eds. Springer, 2021; pp 1–5.

[ref48] Pham N. T. Characterization of Low-Density Polyethylene and LDPE-Based/Ethylene-Vinyl Acetate with Medium Content of Vinyl Acetate. Polymers (Basel) 2021, 13, 2352 10.3390/polym13142352. 34301109 PMC8309532

[ref49] Polyethylene production in the United States from 1990 to 2019 (in million metric tons). Statista, 2020. https://www.statista.com/statistics/975591/us-polyethylene-production-volume (accessed 2024-05-07).

[ref50] Total plastic municipal solid waste (MSW) recycled in the United States in 2018, by resin (in 1,000 tons). Statista, 2023. https://www.statista.com/statistics/1228851/us-plastic-waste-recycled-by-resin (accessed 2024-02-15).

[ref51] Pinheiro L. A.; Chinelatto M. A.; Canevarolo S. V. The role of chain scission and chain branching in high density polyethylene during thermo-mechanical degradation. Polym. Degrad. Stab. 2004, 86, 445–453. 10.1016/j.polymdegradstab.2004.05.016.

[ref52] Boldizar A.; Jansson A.; Gevert T.; Möller K. Simulated recycling of post-consumer high density polyethylene material. Polym. Degrad. Stab. 2000, 68, 317–319. 10.1016/S0141-3910(00)00012-4.

[ref53] Bitting D.; Parker K. R.; Polasek M. G.; Slivensky D. E.; Wu X. Processes and systems for making recycle content hydrocarbons.US 20210130699 A1, 2021.

[ref54] Timken H. Circular economy for plastic waste to polyethylene via refinery crude unit. US 11174436 B2, 2021.

[ref55] Ramamurthy K. K.; Narayanaswamy R. B.; Bhotla V. R. G.; Stanislaus A.; Ganji S. Conversion of waste plastic to propylene and cumene. US 10851309 B2, 2020.

[ref56] Ramamurthy K. K.; Narayanaswamy R.; Bhotla V. R. G.; Stanislaus A.; Ganji S. Conversion of waste plastic through pyrolysis to high value products like benzene and xylenes. US 10975313 B2, 2021.

[ref57] Scheibel J. J.; Vinson P. K.; Cron S. L.; Williams T. E. Methods for producing alkylbenzenes, paraffins, olefins and oxo alcohols from waste plastic feedstocks. US 20210108154 A1, 2021.

[ref58] Kumar A.; Kumar P. Catalytic depolymerization of polymeric materials. US 10457886 B2, 2019.

[ref59] Musa A.; Jaseer E. A.; Barman S.; Garcia N. Review on Catalytic Depolymerization of Polyolefin Waste by Hydrogenolysis: State-of-the-Art and Outlook. Energy Fuels 2024, 38, 1676–1691. 10.1021/acs.energyfuels.3c04109.

[ref60] Zhang F.; Zeng M.; Yappert R. D.; Sun J.; Lee Y.-H.; LaPointe A. M.; Peters B.; Abu-Omar M. M.; Scott S. L. Polyethylene upcycling to long-chain alkylaromatics by tandem hydrogenolysis/aromatization. Science 2020, 370, 437–441. 10.1126/science.abc5441. 33093105

[ref61] Hu P.; Zhang C.; Chu M.; Wang X.; Wang L.; Li Y.; Yan T.; Zhang L.; Ding Z.; Cao M.; Xu P.; Li Y.; Cui Y.; Zhang Q.; Chen J.; Chi L. Stable Interfacial Ruthenium Species for Highly Efficient Polyolefin Upcycling. J. Am. Chem. Soc. 2024, 146, 7076–7087. 10.1021/jacs.4c00757. 38428949

[ref62] Sullivan K. P.; Werner A. Z.; Ramirez K. J.; Ellis L. D.; Bussard J. R.; Black B. A.; Brandner D. G.; Bratti F.; Buss B. L.; Dong X.; Haugen S. J.; Ingraham M. A.; Konev M. O.; Michener W. E.; Miscall J.; Pardo I.; Woodworth S. P.; Guss A. M.; Román-Leshkov Y.; Stahl S. S.; Beckham G. T. Mixed plastics waste valorization through tandem chemical oxidation and biological funneling. Science 2022, 378, 207–211. 10.1126/science.abo4626. 36227984

[ref63] Rabot C.; Chen Y.; Bijlani S.; Chiang Y.-M.; Oakley C. E.; Oakley B. R.; Williams T. J.; Wang C. C. C. Conversion of Polyethylenes into Fungal Secondary Metabolites. Angew. Chem., Int. Ed. 2023, 62, e202214609 10.1002/anie.202214609. PMC10100090 36417558

[ref64] Wang K.; Jia R.; Cheng P.; Shi L.; Wang X.; Huang L. Highly Selective Catalytic Oxi-upcycling of Polyethylene to Aliphatic Dicarboxylic Acid under a Mild Hydrogen-Free Process. Angew. Chem., Int. Ed. 2023, 62, e202301340 10.1002/anie.202301340. 37211533

[ref65] Conk R. J.; Hanna S.; Shi J. X.; Yang J.; Ciccia N. R.; Qi L.; Bloomer B. J.; Heuvel S.; Wills T.; Su J.; Bell A. T.; Hartwig J. F. Catalytic deconstruction of waste polyethylene with ethylene to form propylene. Science 2022, 377, 1561–1566. 10.1126/science.add1088. 36173865 PMC11723507

[ref66] Wang N. M.; Strong G.; DaSilva V.; Gao L.; Huacuja R.; Konstantinov I. A.; Rosen M. S.; Nett A. J.; Ewart S.; Geyer R.; Scott S. L.; Guironnet D. Chemical Recycling of Polyethylene by Tandem Catalytic Conversion to Propylene. J. Am. Chem. Soc. 2022, 144, 18526–18531. 10.1021/jacs.2c07781. 36178850

[ref67] Polyvinyl chloride (PVC) production volume worldwide in 2018 and 2025 (in million metric tons). Statista, 2018. https://www.statista.com/statistics/720296/global-polyvinyl-chloride-market-size-in-tons/(accessed 2024-02-16).

[ref68] Yu J.; Sun L.; Ma C.; Qiao Y.; Yao H. Thermal degradation of PVC: A review. Waste Manage. 2016, 48, 300–314. 10.1016/j.wasman.2015.11.041. 26687228

[ref69] Allsopp M. W.; Vianello G. Poly(Vinyl Chloride). In Ullmann’s Encyclopedia of Industrial Chemistry; Wiley-VCH Verlag GmbH & Co., 2000; pp 441–467.

[ref70] Khopade K. V.; Chikkali S. H.; Barsu N. Metal-catalyzed plastic depolymerization. Cell Rep. Phys. Sci. 2023, 4, 101341 10.1016/j.xcrp.2023.101341.

[ref71] Lewandowski K.; Skórczewska K. A Brief Review of Poly(Vinyl Chloride) (PVC) Recycling. Polymers (Basel) 2022, 14, 3035 10.3390/polym14153035. 35893999 PMC9332854

[ref72] Sadat-Shojai M.; Bakhshandeh G.-R. Recycling of PVC wastes. Polym. Degrad. Stab. 2011, 96, 404–415. 10.1016/j.polymdegradstab.2010.12.001.

[ref73] Brown K. A.; Holland M.; Boyd R.; Thresh S.; Jones H.; Ogilvie S. Economic evaluation of PVC waste management. AEA Technology: 1998.

[ref74] PVC recycling technologies; VinylPlus, 2017. https://vinylplus.eu/documents/6/60/PVC-recycling-technologies (accessed on 2024-02-18).

[ref75] Okuwaki A. Feedstock recycling of plastics in Japan. Polym. Degrad. Stab. 2004, 85, 981–988. 10.1016/j.polymdegradstab.2004.01.023.

[ref76] Yamamoto T.; Sato H.; Matsukura Y.; Kasai A.; Ujisawa Y.; Ishida H.; Kikuchi R. High efficiency heat and material recovery of plastic waste including polyvinyl chloride by using Sumitomo Metals’ waste gasification and smelting system. In Sustainable Development of Energy, Water and Environment Systems; World Scientific, 2007; pp 536–547.

[ref77] O’Rourke G.; Hennebel T.; Stalpaert M.; Skorynina A.; Bugaev A.; Janssens K.; Van Emelen L.; Lemmens V.; De Oliveira Silva R.; Colemonts C.; Gabriels P.; Sakellariou D.; De Vos D. Catalytic tandem dehydrochlorination–hydrogenation of PVC towards valorisation of chlorinated plastic waste. Chem. Sci. 2023, 14, 4401–4412. 10.1039/D3SC00945A. 37123179 PMC10132168

[ref78] Buhl R. Progress in PVC feedstock recycling. Polimery 2003, 48, 263–267. 10.14314/polimery.2003.263.

[ref79] Wigand M.; Bowman L.; Weineke M.; Muller R. Method for the industrial production of calcium carbide in an electric low-body shaft furnace. CN 101883746 A, 2010.

[ref80] Zhang Z.; Peng H.; Yang D.; Zhang G.; Zhang J.; Ju F. Polyvinyl chloride degradation by a bacterium isolated from the gut of insect larvae. Nat. Commun. 2022, 13, 5360 10.1038/s41467-022-32903-y. 36097154 PMC9468159

[ref81] Peng B.-Y.; Chen Z.; Chen J.; Yu H.; Zhou X.; Criddle C. S.; Wu W.-M.; Zhang Y. Biodegradation of Polyvinyl Chloride (PVC) in Tenebrio molitor (Coleoptera: Tenebrionidae) larvae. Environ. Int. 2020, 145, 106106 10.1016/j.envint.2020.106106. 32947161

[ref82] Skelly P. W.; Chang C. F.; Braslau R. Degradation of Polyvinyl Chloride by Sequential Dehydrochlorination and Olefin Metathesis. ChemPlusChem. 2023, 88, e202300184 10.1002/cplu.202300184. 37100741

[ref83] Jiao X.; Zheng K.; Chen Q.; Li X.; Li Y.; Shao W.; Xu J.; Zhu J.; Pan Y.; Sun Y.; Xie Y. Photocatalytic Conversion of Waste Plastics into C_2_ Fuels under Simulated Natural Environment Conditions. Angew. Chem., Int. Ed. 2020, 59, 15497–15501. 10.1002/anie.201915766. 32003512

[ref84] Maddah H. A. Polypropylene as a Promising Plastic: A Review. Am. J. Polym. Sci. 2016, 6, 1–11.

[ref85] Alsabri A.; Tahir F.; Al-Ghamdi S. G. Environmental impacts of polypropylene (PP) production and prospects of its recycling in the GCC region. Mater. Today: Proc. 2022, 56, 2245–2251. 10.1016/j.matpr.2021.11.574.

[ref86] Production volume of polypropylene resin worldwide in 2018 and 2026 (in million metric tons). Statista, 2019. https://www.statista.com/statistics/1103529/global-polypropylene-production/ (accessed 2024-02-19).

[ref87] Gahleitner M.; Paulik C. Polypropylene. Ullmann’s Encyclopedia of Industrial Chemistry 2014, 1–44. 10.1002/14356007.o21_o04.pub2.

[ref88] US EPA Advancing Sustainable Materials Management: 2016 and 2017 Tables and Figures; US Environmental Protection Agency, 2019. https://www.epa.gov/sites/production/files/2019-11/documents/2016_and_2017_facts_and_figures_data_tables_0.pdf (accessed 2024-02-19).

[ref89] Schyns Z. O. G.; Shaver M. P. Mechanical Recycling of Packaging Plastics: A Review. Macromol. Rapid Commun. 2021, 42, 2000415 10.1002/marc.202000415. 33000883

[ref90] Bora R. R.; Wang R.; You F. Waste Polypropylene Plastic Recycling toward Climate Change Mitigation and Circular Economy: Energy, Environmental, and Technoeconomic Perspectives. ACS Sus. Chem. Eng. 2020, 8, 16350–16363. 10.1021/acssuschemeng.0c06311.

[ref91] Daria F., Chemical Recycling of Polyolefins (PE, PP): Modern Technologies and Products. In Waste Material Recycling in the Circular Economy; Dimitris S. A., Ed.; IntechOpen, 2021; p Ch. 4.

[ref92] Badiola C.; Wang S.; Karri R.; Findlay J. Apparatus and processes for pyrolysis of plastic feeds. WO 2023279016 A1, 2023.

[ref93] Encina announces $1.8 billion investment in new advanced manufacturing facility in Pennsylvania. Encina, 2022. https://www.encina.com/encina-announces-1-1-billion-investment-in-new-advanced-manufacturing-facility-in-pennsylvania/(accessed 2024-02-19).

[ref94] Gold J. W.; Cudney C. Hydrocarbon compositions derived from pyrolysis of post-consumer and/or post-industrial plastics and methods of making ad use thereof. US 11891518 B1, 2024.

[ref95] Rorrer J. E.; Troyano-Valls C.; Beckham G. T.; Román-Leshkov Y. Hydrogenolysis of Polypropylene and Mixed Polyolefin Plastic Waste over Ru/C to Produce Liquid Alkanes. ACS Sus. Chem. Eng. 2021, 9, 11661–11666. 10.1021/acssuschemeng.1c03786.

[ref96] Kots P. A.; Liu S.; Vance B. C.; Wang C.; Sheehan J. D.; Vlachos D. G. Polypropylene Plastic Waste Conversion to Lubricants over Ru/TiO2 Catalysts. ACS Catal. 2021, 11, 8104–8115. 10.1021/acscatal.1c00874.

[ref97] Dong Q.; Lele A. D.; Zhao X.; Li S.; Cheng S.; Wang Y.; Cui M.; Guo M.; Brozena A. H.; Lin Y.; Li T.; Xu L.; Qi A.; Kevrekidis I. G.; Mei J.; Pan X.; Liu D.; Ju Y.; Hu L. Depolymerization of plastics by means of electrified spatiotemporal heating. Nature 2023, 616, 488–494. 10.1038/s41586-023-05845-8. 37076729

[ref98] Xu Z.; Munyaneza N. E.; Zhang Q.; Sun M.; Posada C.; Venturo P.; Rorrer N. A.; Miscall J.; Sumpter B. G.; Liu G. Chemical upcycling of polyethylene, polypropylene, and mixtures to high-value surfactants. Science 2023, 381, 666–671. 10.1126/science.adh0993. 37561876

[ref99] Scheirs J.; Priddy D. Historical Overview of Styrenic Polymers. Modern Styrenic Polymers: Polystyrenes and Styrenic Copolymers. John Wiley & Sons, 2003.

[ref100] Production capacity of polystyrene worldwide in 2022 and 2026 (in million metric tons). Statista, 2023. https://www.statista.com/statistics/1065889/global-polystyrene-production-capacity (accessed 2024-02-20).

[ref101] Maul J.; Frushour B. G.; Kontoff J. R.; Eichenauer H.; Ott K.-H.; Schade C., Polystyrene and Styrene Copolymers. In Ullmann’s Encyclopedia of Industrial Chemistry; Wiley-VCH Verlag GmbH & Co., 2007; pp 475–522.

[ref102] Capricho J. C.; Prasad K.; Hameed N.; Nikzad M.; Salim N. Upcycling Polystyrene. Polymers 2022, 14, 5010 10.3390/polym14225010. 36433142 PMC9695542

[ref103] First commercial polystyrene recycling plant. Polystyvert, 2023. https://polystyvert.com/en/accelerated-growth-for-polystyvert (accessed 2024-02-21).

[ref104] New milestone towards a circular economy for the PS industry. PS Loop, 2024. https://www.psloop.eu/news/new_milestone (2024-02-21).

[ref105] Cavinaw B.; Crawford S.; Lamaze S.; Allen D.; Christenson E.; Rumford M. Systems and methods for recycling waste plastics, including waste polystyrene. US 11466215 B2, 2021.

[ref106] Maisonneuve L.; Mathieu F. Process for depolymerization of a polystyrenic filler by pyrolysis. FR 3137384 A1, 2024.

[ref107] Benecke B.; Niessner N.; Kerschbaumer H.; Abboud M. Recycling method for styrene-containing plastic waste. WO 2018224482 A1, 2018.

[ref108] Kanattukara B. V.; Singh G.; Singh D.; Kapur G. S.; Ramakumar S. S. V. Catalytic pyrolysis of polystyrene into aromatic rich liquid product using spherical catalyst. EP 3971264 A1, 2022.

[ref109] Tippet J.; Butler J.; Assef J.; Ashbaugh J.; Clark J.; Duc M.; Cary J.-B. Depolymerization of plastic materials. US 9650313 B2, 2017.

[ref110] Agilyx and INEOS Styrolution advance development of large scale TruStyrenyx plant. Agilyx, 2023. https://www.agilyx.com/agilyx-and-ineos-styrolution-advance-development-of-large-scale-trustyrenyx-plant/(accessed 2024-02-21).

[ref111] Depolymerisation: Moving from lab-scale to pilot-scale. INEOS Styrolution. https://styrolution-eco.com/depolymerisation-moving-from-lab-scale-to-pilot-scale.html (accessed 2024-02-21).

[ref112] IndianOil board accords Stage-1 approval for setting up the first-ever styrene project in India with a CAPEX of Rs 4495 crore. IndianOil, 2021. https://iocl.com/NewsDetails/59272 (accessed 2024-02-21).

[ref113] Wang J.; Ma Y.; Li S.; Yue C. Catalytic pyrolysis of polystyrene in different reactors: Effects of operating conditions on distribution and composition of products. J. Anal. Appl. Pyrolysis 2024, 177, 106366 10.1016/j.jaap.2024.106366.

[ref114] Oh S.; Stache E. E. Chemical Upcycling of Commercial Polystyrene via Catalyst-Controlled Photooxidation. J. Am. Chem. Soc. 2022, 144, 5745–5749. 10.1021/jacs.2c01411. 35319868

[ref115] Huang Z.; Shanmugam M.; Liu Z.; Brookfield A.; Bennett E. L.; Guan R.; Vega Herrera D. E.; Lopez-Sanchez J. A.; Slater A. G.; McInnes E. J. L.; Qi X.; Xiao J. Chemical Recycling of Polystyrene to Valuable Chemicals via Selective Acid-Catalyzed Aerobic Oxidation under Visible Light. J. Am. Chem. Soc. 2022, 144, 6532–6542. 10.1021/jacs.2c01410. 35353526 PMC9011358

[ref116] Xu S.; Liu S.; Song W.; Zheng N. Metal-free upcycling of plastic waste: photo-induced oxidative degradation of polystyrene in air. Green Chem. 2024, 26, 1363–1369. 10.1039/D3GC04197B.

[ref117] Xu Z.; Pan F.; Sun M.; Xu J.; Munyaneza N. E.; Croft Z. L.; Cai G.; Liu G. Cascade degradation and upcycling of polystyrene waste to high-value chemicals. Proc. Natl. Acad. Sci. U.S.A. 2022, 119, e2203346119 10.1073/pnas.2203346119. 35969757 PMC9407675

[ref118] Sponchioni M.; Altinok S. Poly(methyl methacrylate): Market trends and recycling. Adv. Chem. Eng. 2022, 60, 269–287. 10.1016/bs.ache.2022.09.004.

[ref119] Stickler M.; Rhein T. Polymethacrylates. In Ullmann’s Encyclopedia of Industrial Chemistry; Wiley-VCH Verlag GmbH & Co., 2000; pp 341–353.

[ref120] Esmizadeh E.; Khalili S.; Vahidifar A.; Naderi G.; Dubois C. Waste Polymethyl Methacrylate (PMMA): Recycling and High-Yield Monomer Recovery. In Handbook of Ecomaterials; Martínez L. M. T., Kharissova O. V., Kharisov B. I., Eds.; Springer, 2018; pp 1–33.

[ref121] Faris T. V.; Gordon G. T.; Chan D. T. High heat distortion temperature methacrylate polymer blends. US 6653405 B2, 2003.

[ref122] Weiss H.-J.; Schmalfeld J.; Zentner U.; Groschang T.; Gropp U.; Fuss W.; Goedecke R.; Schöla E. Method for depolymerizing polymethylmethacrylate. US 6469203 B1, 2002.

[ref123] Dubois J.-L.; Van der Heijden S. Process for recycling contaminated polymers. EP 4206267 A1, 2021.

[ref124] Sasaki A.; Kikuya N.; Ookubo T.; Hayashida M. Recovery method of pyrolysis product of resin. US 8304573 B2, 2012.

[ref125] Godiya C. B.; Gabrielli S.; Materazzi S.; Pianesi M. S.; Stefanini N.; Marcantoni E. Depolymerization of waste poly(methyl methacrylate) scraps and purification of depolymerized products. J. Environ. Manag. 2019, 231, 1012–1020. 10.1016/j.jenvman.2018.10.116. 30602225

[ref126] Mitsubishi Chemical and Agilyx announce successful trial results for advanced recycling collaboration. Agilyx, 2021. https://www.agilyx.com/mitsubishi-chemical-and-agilyx-announce-successful-trial-results-for-advanced-recycling-collaboration-2 (accessed 2024-02-22).

[ref127] New Trinseo PMMA depolymerization plant reimagines plastics value chain with sustainability in mind. Trinseo, 2023. https://investor.trinseo.com/home/news/news-details/2023/New-Trinseo-PMMA-Depolymerization-Plant-Reimagines-Plastics-Value-Chain-With-Sustainability-In-Mind (accessed 2024-02-22).

[ref128] Sumitomo Chemical completes construction of pilot facility for acrylic resin chemical recycling. Sumitomo Chemical, 2022. https://www.sumitomo-chem.co.jp/english/news/detail/20221223e.html (accessed 2024-02-22).

[ref129] Kugelmass L. H.; Tagnon C.; Stache E. E. Photothermal Mediated Chemical Recycling to Monomers via Carbon Quantum Dots. J. Am. Chem. Soc. 2023, 145, 16090–16097. 10.1021/jacs.3c04448. 37432654

[ref130] Jones G. R.; Wang H. S.; Parkatzidis K.; Whitfield R.; Truong N. P.; Anastasaki A. Reversed Controlled Polymerization (RCP): Depolymerization from Well-Defined Polymers to Monomers. J. Am. Chem. Soc. 2023, 145, 9898–9915. 10.1021/jacs.3c00589. 37127289 PMC10176471

[ref131] Wang H. S.; Truong N. P.; Pei Z.; Coote M. L.; Anastasaki A. Reversing RAFT Polymerization: Near-Quantitative Monomer Generation Via a Catalyst-Free Depolymerization Approach. J. Am. Chem. Soc. 2022, 144, 4678–4684. 10.1021/jacs.2c00963. 35213149 PMC8931752

[ref132] Parkatzidis K.; Wang H. S.; Anastasaki A. Photocatalytic Upcycling and Depolymerization of Vinyl Polymers. Angew. Chem., Int. Ed. 2024, 63, e202402436 10.1002/anie.202402436. 38466624

[ref133] De Luca Bossa F.; Yilmaz G.; Matyjaszewski K. Fast Bulk Depolymerization of Polymethacrylates by ATRP. ACS Macro Lett. 2023, 12, 1173–1178. 10.1021/acsmacrolett.3c00389. 37531639 PMC10433507

[ref134] Young J. B.; Hughes R. W.; Tamura A. M.; Bailey L. S.; Stewart K. A.; Sumerlin B. S. Bulk depolymerization of poly(methyl methacrylate) via chain-end initiation for catalyst-free reversion to monomer. Chem. 2023, 9, 2669–2682. 10.1016/j.chempr.2023.07.004.

[ref135] Whitfield R.; Jones G. R.; Truong N. P.; Manring L. E.; Anastasaki A. Solvent-Free Chemical Recycling of Polymethacrylates made by ATRP and RAFT polymerization: High-Yielding Depolymerization at Low Temperatures. Angew. Chem., Int. Ed. 2023, 62, e202309116 10.1002/anie.202309116. 37523176

[ref136] Chin M. T.; Yang T.; Quirion K. P.; Lian C.; Liu P.; He J.; Diao T. Implementing a Doping Approach for Poly(methyl methacrylate) Recycling in a Circular Economy. J. Am. Chem. Soc. 2024, 146, 5786–5792. 10.1021/jacs.3c13223. 38382057 PMC10921398

[ref137] Hughes R. W.; Lott M. E.; Zastrow I. S.; Young J. B.; Maity T.; Sumerlin B. S. Bulk Depolymerization of Methacrylate Polymers via Pendent Group Activation. J. Am. Chem. Soc. 2024, 146, 6217–6224. 10.1021/jacs.3c14179. 38382047

[ref138] Martinez M. R.; Schild D.; De Luca Bossa F.; Matyjaszewski K. Depolymerization of Polymethacrylates by Iron ATRP. Macromolecules 2022, 55, 10590–10599. 10.1021/acs.macromol.2c01712.

[ref139] Serini V. Polycarbonates. In Ullmann’s Encyclopedia of Industrial Chemistry; Wiley-VCH Verlag GmbH & Co., 2000; pp 603–611.

[ref140] Fukuoka S.; Fukawa I.; Adachi T.; Fujita H.; Sugiyama N.; Sawa T. Industrialization and Expansion of Green Sustainable Chemical Process: A Review of Non-phosgene Polycarbonate from CO_2_. Org. Process Res. Dev. 2019, 23, 145–169. 10.1021/acs.oprd.8b00391.

[ref141] Kumar A. V. A.; Pothuraju K.; Lakshmi K. R.; Bhowmik S. Development of light weight transparent bullet proof polymeric laminates. Polym. Eng. Sci. 2023, 63, 3698–3707. 10.1002/pen.26477.

[ref142] den Braver-Sewradj S. P.; van Spronsen R.; Hessel E. V. S. Substitution of bisphenol A: a review of the carcinogenicity, reproductive toxicity, and endocrine disruption potential of alternative substances. Crit. Rev. Toxicol. 2020, 50, 128–147. 10.1080/10408444.2019.1701986. 32031044

[ref143] Production of polycarbonates worldwide by region in 2016 (in million metric tons). Statista, 2017.https://www.statista.com/statistics/650318/polycarbonates-production-worldwide-and-in-europe (accessed 2024-02-23).

[ref144] Antonakou E. V.; Achilias D. S. Recent Advances in Polycarbonate Recycling: A Review of Degradation Methods and Their Mechanisms. Waste Biomass Valori 2013, 4 (1), 9–21. 10.1007/s12649-012-9159-x.

[ref145] Eiden S.; Bellinghausen R.; Wolf A.; Sluyts E.; Krause J.; Seidel A. Pyrolysis of polycarbonate-containing material in order to recover raw materials. EP 4294892 A1, 2023.

[ref146] Eiden S.; Bellinghausen R.; Wolf A.; Sluyts E.; Krause J.; Seidel A. Pyrolysis of polycarbonate-containing material in order to recover raw materials. WO 2022174963 A1, 2022.

[ref147] Kikuya N.; Okubu T. Thermal decomposition device and thermal decomposition method of resin. JP 2011236337 A, 2011.

[ref148] Emre-Flender S. Chemical recycling of polycarbonates reaches a major milestone. Covestro, 2023. https://www.covestro.com/press/chemical-recycling-of-polycarbonates-reaches-a-major-milestone (accessed 2024-02-25).

[ref149] Study on the World’s First Commercialization of Polycarbonate Resin Chemical Recycling. Mitsubishi Chemical Group, 2023. https://www.mcgc.com/english/news_release/01528.html (accessed 2024-02-25).

[ref150] Mahood J. A.; Gorman J. L. III; Pingitore A. T.; Scales C. E.; Shankwitz G. P. Depolymerization of a poly(carbonate) and isolation of bisphenol a from a depolymerized poly(carbonate). WO 2020257237 A1, 2020.

[ref151] Kithara M.; Hirata M.; Ban T.; Sawaki T. Method of decomposing polycarbonate. US 7585930 B2, 2009.

[ref152] Hironaka K.; Miyake R.; Inomata H.; Watanabe M. Methods for recovering bisphenol compound. JP 2008195646 A, 2008.

[ref153] Kim J. G. Chemical recycling of poly(bisphenol A carbonate). Polym. Chem. 2020, 11, 4830–4849. 10.1039/C9PY01927H.

[ref154] Westhues S.; Idel J.; Klankermayer J. Molecular catalyst systems as key enablers for tailored polyesters and polycarbonate recycling concepts. Sci. Adv. 2018, 4, eaat9669 10.1126/sciadv.aat9669. 30105308 PMC6086616

[ref155] Alberti C.; Eckelt S.; Enthaler S. Ruthenium-Catalyzed Hydrogenative Depolymerization of End-of-Life Poly(bisphenol A carbonate). ChemistrySelect 2019, 4, 12268–12271. 10.1002/slct.201903549. PMC6905177 31867148

[ref156] Kindler T.-O.; Alberti C.; Sundermeier J.; Enthaler S. Hydrogenative Depolymerization of End-of-Life Poly-(Bisphenol A Carbonate) Catalyzed by a Ruthenium-MACHO-Complex. ChemistryOpen 2019, 8, 1410–1412. 10.1002/open.201900319. 31867148 PMC6905177

[ref157] Estes L. L.; Schweizer M. Fibers, 4. Polyamide Fibers. In Ullmann’s Encyclopedia of Industrial Chemistry; Wiley-VCH Verlag GmbH & Co., 2011; pp 1–14.

[ref158] Herzog B.; Kohan M. I.; Mestemacher S. A.; Pagilagan R. U.; Redmond K.; Sarbandi R., Polyamides. In Ullmann’s Encyclopedia of Industrial Chemistry; Wiley-VCH Verlag GmbH & Co., 2020; pp 1–47.

[ref159] Gupta S., Nylon Polymerization. In Handbook of Polymer Science and Technology, Vol. 1; CRC Press, 1989; pp 211–245.

[ref160] Tonsi G.; Maesani C.; Alini S.; Ortenzi M. A.; Pirola C. Nylon Recycling Processes: a Brief Overview. Chem. Eng. Trans. 2023, 100, 727–732. 10.3303/CET23100122.

[ref161] Corbin T.F.; Davis E. A.; Dellinger J. A. Reclaiming e-caprolactam from nylon 6 carpet. US 5169870 A, 1992.

[ref162] Bassler P.; Kopietz M. Process for producing caprolactam through hydrolytic cleavage of molten polycaprolactam. US 5495015 A, 1996.

[ref163] Moran E. F. Jr. Depolymerization of nylon 6,6 (and optionally) nylon 6 to obtain hexamethylene diamine (and caprolactam). AU 672062 B2, 1996.

[ref164] Nishimura M.; Yamashita K.; Kato K.; Utazaki K. Method for producing recycled polyamide 6 resin composition. JP 2023108237 A, 2023.

[ref165] Sifniades S.; Levy B. A.; Hendrix A. J. Method for depolymerizing waste containing nylon to caprolactam with overheated water vapor in the absence of catalysts. DE 69624378 T2, 2016.

[ref166] Witte D.; Safrit B. T. Method and device for the continuous or batchwise depolymerization of polycaprolactam. CH 700545 A2, 2010.

[ref167] Verduyckt J.; Groothaert M. H. L.; Cuiper A. D.; Fu W.; Tinge J. T. Process for the recovery of epsilon-caprolactam from nylon 6-containing multi-component material. WO 2023144337 A1, 2023.

[ref168] Toray and Honda start jointly validating chemical Nylon 6 recycling for automotive applications. Toray, 2023. https://www.toray.com/global/news/details/20230914101438.html (accessed 2024-02-26).

[ref169] Asahi Kasei and Microwave Chemical launch joint demonstration project for chemical recycling of polyamide 66 using microwave-based technology. Asahi Kasei, 2023. https://www.asahi-kasei.com/news/2023/e230427.html (accessed 2024-02-26).

[ref170] Kumar A.; von Wolff N.; Rauch M.; Zou Y.-Q.; Shmul G.; Ben-David Y.; Leitus G.; Avram L.; Milstein D. Hydrogenative Depolymerization of Nylons. J. Am. Chem. Soc. 2020, 142, 14267–14275. 10.1021/jacs.0c05675. 32706584 PMC7441490

[ref171] Wursthorn L.; Beckett K.; Rothbaum J. O.; Cywar R. M.; Lincoln C.; Kratish Y.; Marks T. J. Selective Lanthanide-Organic Catalyzed Depolymerization of Nylon-6 to ϵ-Caprolactam. Angew. Chem., Int. Ed. 2023, 62, e202212543 10.1002/anie.202212543. 36441664

[ref172] Ye L.; Liu X.; Beckett K. B.; Rothbaum J. O.; Lincoln C.; Broadbelt L. J.; Kratish Y.; Marks T. J. Catalyst metal-ligand design for rapid, selective, and solventless depolymerization of Nylon-6 plastics. Chem. 2024, 10, 172–189. 10.1016/j.chempr.2023.10.022.

[ref173] Moretto H.-H.; Schulze M.; Wagner G., Silicones. In Ullmann’s Encyclopedia of Industrial Chemistry; Wiley-VCH Verlag GmbH & Co., 2000; pp 675–708.

[ref174] Wolf M. P.; Salieb-Beugelaar G. B.; Hunziker P. PDMS with designer functionalities—Properties, modifications strategies, and applications. Prog. Polym. Sci. 2018, 83, 97–134. 10.1016/j.progpolymsci.2018.06.001.

[ref175] Fromme H., Cyclic Volatile Methylsiloxanes: Occurrence and Exposure. In Encyclopedia of Environmental Health, 2nd ed.; Nriagu J., Ed.; Elsevier, 2019; pp 805–812.

[ref176] Buddhima R., Recycling Silicone-Based Materials: An Overview of Methods. In Application and Characterization of Rubber Materials; Gülşen Akın E., Önder P., Eds.; IntechOpen, 2022.

[ref177] Weitkamp R. F.; Neumann B.; Stammler H.-G.; Hoge B. Synthesis and Reactivity of the First Isolated Hydrogen-Bridged Silanol–Silanolate Anions. Angew. Chem., Int. Ed. 2020, 59, 5494–5499. 10.1002/anie.201914339. PMC7154667 31833629

[ref178] Brook M. A.; Zhao S.; Liu L.; Chen Y. Surface etching of silicone elastomers by depolymerization. Can. J. Chem. 2012, 90, 153–160. 10.1139/v11-145.

[ref179] Allandrieu C.; Cardinaud D. Process for the manufacture of cyclosiloxanes by depolymerization of polysiloxanes. US 5670689 A, 1997.

[ref180] Chang C.-L.; Lee H. S.-J.; Chen C.-K. Nucleophilic Cleavage of Crosslinked Polysiloxanes to Cyclic Siloxane Monomers: Mild Catalysis by a Designed Polar Solvent System. J. Polym. Res. 2005, 12, 433–438. 10.1007/s10965-004-1871-1.

[ref181] Giuliani J. R.; Gjersing E. L.; Chinn S. C.; Jones T. V.; Wilson T. S.; Alviso C. T.; Herberg J. L.; Pearson M. A.; Maxwell R. S. Thermal Degradation in a Trimodal Poly(dimethylsiloxane) Network Studied by 1H Multiple Quantum NMR. J. Phys. Chem. B 2007, 111, 12977–12984. 10.1021/jp075840f. 17958412

[ref182] Stary F.; Beisswenger H.; Knies W.; Vogl G.; Scheler D. Continuous thermal degradation of organopolysiloxanes. EP 0748809 A1, 1996.

[ref183] Julian D. J.; Katsoulis D.; Link B. A.; Peitz T. A.; Zhu B. Method of recycling silicone waste with the use of organic polymer and depolymerization catalyst. WO 2014130948 A1, 2014.

[ref184] Advancing silicone recycling for a circular future. Dow, n.d. https://engage.dow.com/LP=13051?linkId=100000225795457 (accessed 2024-03-01).

[ref185] Knott W.; Dudzik H.; Schaefer D. Process for recycling silicones. US 11286366 B2, 2022.

[ref186] Knott W.; Dudzik H.; Schaefer D. Upcycling process for processing silicone wastes. US 11732092 B2, 2023.

[ref187] Rimboeck K.-H.; Mautner K. Recycling of materials containing organosilicon compounds. US 20240010501 A1, 2024.

[ref188] Weitkamp R. F.; Neumann B.; Stammler H.-G.; Hoge B. Synthesis and Reactivity of the First Isolated Hydrogen-Bridged Silanol–Silanolate Anions. Angew. Chem., Int. Ed. 2020, 59, 5494–5499. 10.1002/anie.201914339. PMC7154667 31833629

[ref189] Vu N. D.; Boulègue-Mondière A.; Durand N.; Raynaud J.; Monteil V. Back-to-cyclic monomers: chemical recycling of silicone waste using a [polydentate ligand–potassium silanolate] complex. Green Chem. 2023, 25, 3869–3877. 10.1039/D3GC00293D.

[ref190] Rupasinghe B.; Furgal J. C. Full Circle Recycling of Polysiloxanes via Room-Temperature Fluoride-Catalyzed Depolymerization to Repolymerizable Cyclics. ACS Appl. Polym. Mater. 2021, 3, 1828–1839. 10.1021/acsapm.0c01406.

[ref191] Klose L.; Meyer-Heydecke N.; Wongwattanarat S.; Chow J.; Pérez García P.; Carré C.; Streit W.; Antranikian G.; Romero A. M.; Liese A. Towards Sustainable Recycling of Epoxy-Based Polymers: Approaches and Challenges of Epoxy Biodegradation. Polymers (Basel) 2023, 15, 2653 10.3390/polym15122653. 37376299 PMC10305103

[ref192] Jin F.-L.; Li X.; Park S.-J. Synthesis and application of epoxy resins: A review. J. Ind. Eng. Chem. 2015, 29, 1–11. 10.1016/j.jiec.2015.03.026.

[ref193] Li Y.; Wu Y.; Li K.; Lin H.; Wang M.; Zheng L.; Wu C.; Zhang X. Recycling of Epoxy Resins with Degradable Structures or Dynamic Cross-Linking Networks: A Review. Ind. Eng. Chem. Res. 2024, 63, 5005–5027. 10.1021/acs.iecr.3c03897.

[ref194] Wu Y.; Ge Z.; Huang C.; Zha Z.; Zeng M.; Ma Y.; Sun L.; Hou Z.; Chu S.; Zhang H. In-situ pyrolysis kinetic analysis and fixed-bed pyrolysis behavior of ex-service wind turbine blades. Waste Manag 2023, 168, 54–62. 10.1016/j.wasman.2023.05.049. 37276634

[ref195] Ahrens A.; Bonde A.; Sun H.; Wittig N. K.; Hammershøj H. C. D.; Batista G. M. F.; Sommerfeldt A.; Frølich S.; Birkedal H.; Skrydstrup T. Catalytic disconnection of C–O bonds in epoxy resins and composites. Nature 2023, 617, 730–737. 10.1038/s41586-023-05944-6. 37100913 PMC10208972

[ref196] Liao Y.; Takahashi K.; Nozaki K. Nickel-Catalyzed C(sp^3^)–O Hydrogenolysis via a Remote-Concerted Oxidative Addition and Its Application to Degradation of a Bisphenol A-Based Epoxy Resin. J. Am. Chem. Soc. 2024, 146, 2419–2425. 10.1021/jacs.3c09061. 38060439

[ref197] Sun H.; Ahrens A.; Batista G. M. F.; Donslund B. S.; Ravn A. K.; Schwibinger E. V.; Nova A.; Skrydstrup T. Solvent–base mismatch enables the deconstruction of epoxy polymers and bisphenol A recovery. Green Chem. 2024, 26, 815–824. 10.1039/D3GC03707J.

[ref198] DiPucchio R. C.; Stevenson K. R.; Lahive C. W.; Michener W. E.; Beckham G. T. Base-Mediated Depolymerization of Amine-Cured Epoxy Resins. ACS Sus. Chem. Eng. 2023, 11, 16946–16954. 10.1021/acssuschemeng.3c04181. PMC10698742 38076616

[ref199] Nguyen S. T.; Fries L. R.; Cox J. H.; Ma Y.; Fors B. P.; Knowles R. R. Chemical Recycling of Thiol Epoxy Thermosets via Light-Driven C–C Bond Cleavage. J. Am. Chem. Soc. 2023, 145, 11151–11160. 10.1021/jacs.3c00958. 37167410

[ref200] Larder R. R.; Hatton F. L. Enabling the Polymer Circular Economy: Innovations in Photoluminescent Labeling of Plastic Waste for Enhanced Sorting. ACS Polym. Au 2023, 3, 182–201. 10.1021/acspolymersau.2c00040. 37065718 PMC10103190

[ref201] Lange J.-P. Managing Plastic Waste–Sorting, Recycling, Disposal, and Product Redesign. ACS Sus. Chem. Eng. 2021, 9, 15722–15738. 10.1021/acssuschemeng.1c05013.

[ref202] Li C.; Kong X. Y.; Lyu M.; Tay X. T.; Đokić M.; Chin K. F.; Yang C. T.; Lee E. K. X.; Zhang J.; Tham C. Y.; Chan W. X.; Lee W. J.; Lim T. T.; Goto A.; Sullivan M. B.; Soo H. S. Upcycling of non-biodegradable plastics by base metal photocatalysis. Chem 2023, 9, 2683–2700. 10.1016/j.chempr.2023.07.008.

[ref203] García J. M. Catalyst: Design Challenges for the Future of Plastics Recycling. Chem. 2016, 1, 813–815. 10.1016/j.chempr.2016.11.003.

[ref204] Haque F. M.; Ishibashi J. S. A.; Lidston C. A. L.; Shao H.; Bates F. S.; Chang A. B.; Coates G. W.; Cramer C. J.; Dauenhauer P. J.; Dichtel W. R.; Ellison C. J.; Gormong E. A.; Hamachi L. S.; Hoye T. R.; Jin M.; Kalow J. A.; Kim H. J.; Kumar G.; LaSalle C. J.; Liffland S.; Lipinski B. M.; Pang Y.; Parveen R.; Peng X.; Popowski Y.; Prebihalo E. A.; Reddi Y.; Reineke T. M.; Sheppard D. T.; Swartz J. L.; Tolman W. B.; Vlaisavljevich B.; Wissinger J.; Xu S.; Hillmyer M. A. Defining the Macromolecules of Tomorrow through Synergistic Sustainable Polymer Research. Chem. Rev. 2022, 122, 6322–6373. 10.1021/acs.chemrev.1c00173. 35133803

